# The malignancy suppression and ferroptosis facilitation of BCL6 in gastric cancer mediated by FZD7 repression are strengthened by RNF180/RhoC pathway

**DOI:** 10.1186/s13578-023-01020-8

**Published:** 2023-04-14

**Authors:** Shiwei Guo, Jingyu Deng, Pengliang Wang, Fan Kou, Zizhen Wu, Nannan Zhang, Zhenzhen Zhao, Yongzhan Nie, Lili Yang

**Affiliations:** 1 Department of Immunology, Key Laboratory of Cancer Immunology and Biotherapy, Tianjin Medical University Cancer Institute and Hospital, National Clinical Research Center for Cancer, Tianjin’s Clinical Research Center for Cancer, Tianjin, 300060 China; 2 Department of Gastric Surgery, Tianjin Medical University Cancer Institute and Hospital, National Clinical Research Center for Cancer, Tianjin’s Clinical Research Center for Cancer, Tianjin, 300060 China; 3 Department of Gastrointestinal Surgery, Sun Yat-sen Memorial Hospital, Sun Yat-sen University, Guangzhou, 510120 China; 4 State Key Laboratory of Cancer Biology, National Clinical Research Center for Digestive Diseases and Xijing Hospital of Digestive Diseases, The Fourth Military Medical University, Xi’an, 710032 Shaanxi China

**Keywords:** BCL6, Gastric cancer, Proliferation, Metastasis, FZD7, Wnt/β-catenin pathway, GPX4, Ferroptosis, RNF180, RhoC

## Abstract

**Background:**

B-cell lymphoma 6 (BCL6) is a transcription repressor that plays a tumor suppressor or promoting role in various tumors. However, its function and molecular mechanism in gastric cancer (GC) remain unclear. Ferroptosis, a novel programmed cell death, is closely related to tumor development. In this research, we aimed to explore the role and mechanism of BCL6 in malignant progression and ferroptosis of gastric cancer.

**Methods:**

Firstly, BCL6 was identified as an important biomarker that attenuated the proliferation and metastasis of GC through tumor microarrays and confirmed in GC cell lines. RNA sequence was performed to explore the downstream genes of BCL6. The underlying mechanisms were further investigated by ChIP, dual luciferase reporter assays and rescue experiments. Cell death, lipid peroxidation, MDA and Fe^2+^ level were detected to determine the effect of BCL6 on ferroptosis and the mechanism was revealed. CHX, MG132 treatment and rescue experiments were used to explore the upstream regulatory mechanism of BCL6.

**Results:**

Here we showed that BCL6 expression was significantly decreased in GC tissues, and patients with low BCL6 expression showed more malignant clinical features and poor prognosis. The upregulation of BCL6 may significantly inhibited the proliferation and metastasis of GC cells in vitro and in vivo. In addition, we found that BCL6 directly binds and transcriptionally represses Wnt receptor Frizzled 7 (FZD7) to inhibit the proliferation, metastasis of GC cells. We also found that BCL6 promoted lipid peroxidation, MDA and Fe^2+^ level to facilitate ferroptosis of GC cells by FZD7/β-catenin/TP63/GPX4 pathway. Furthermore, the expression and function of BCL6 in GC were regulated by the ring finger protein 180 (RNF180)/ras homolog gene family member C (RhoC) pathway, which had been elucidated to be involved in significantly mediating the proliferation and metastasis of GC cells.

**Conclusions:**

In summary, BCL6 should be considered a potential intermediate tumor suppressor to inhibit the malignant progression and induce ferroptosis, which might be a promising molecular biomarker for further mechanistic investigation of GC.

**Supplementary Information:**

The online version contains supplementary material available at 10.1186/s13578-023-01020-8.

## Introduction

In recent years, molecular-targeted therapies have played an increasingly important role in the treatment of GC. The protein encoded by the B-cell lymphoma 6 (*BCL6*) gene is a transcriptional repressor that suppresses the transcription of target genes [1, 2]. Most germinal center-derived lymphomas depend on the expression and transcriptional activity of BCL6 [3]. With the in-depth study of BCL6 and hematological tumors such as lymphoma in recent years, it has been revealed that BCL6 is closely related to various solid tumors such as breast cancer and lung cancer, and played various roles in different tumors [4–8]. For example, BCL6 overexpression promoted metastasis in lung cancer [5]. Similarly, BCL6 could promote breast cancer metastasis [6]. In medulloblastoma (MB), BCL6 could lead to epigenetic inhibition of the GLI family zinc finger 1/2 (GLI1 and GLI2), which are the critical effectors of the sonic hedgehog (Shh) pathway [7, 9]. In HB-EGF-CTF-positive GC, the ectopic degradation of BCL6 in the cytoplasm promoted the expression of cyclin D2, leading to the acceleration of the cell cycle [8]. Therefore, BCL6 could inhibit the malignant progression of some tumors. Nevertheless, research is limited to HB-EGF-CTF-positive GC, and its mechanism in other patients with GC is poorly understood.

Ferroptosis is a new type of programmed cell death driven by iron-dependent lipid peroxidation. Generally speaking, ferroptosis is closely related to cell redox balance and iron ions, and the main driving factor is the peroxidation of cell membrane phospholipids. The accumulation of lethal dose of lipid peroxides is a major feature of ferroptosis [10, 11]. At present, there are mainly three kinds of ferroptosis defense systems: GPX4-GSH defense system, FSP1-CoQH2 defense system and DHODH-CoQH2 defense system [12–17]. Among them, GPX4-GSH defense system is the most classic ferroptosis antioxidant defense system. Thiol-containing tripeptide glutathione (GSH) is not only the main antioxidant in mammalian cells, but also the primary substrate of GPX4. When glutathione deficiency or GPX4 activity decreased, the antioxidant capacity of cells decreased, resulting in lipid peroxidation and metabolic dysfunction. Finally, lipid- reactive oxygen species (ROS) and MDA accumulated to lethal levels, leading to ferroptosis [12, 18, 19].

In this study, we identified BCL6 as an important transcription repressor that inhibited the malignant progression and facilitates ferroptosis of gastric cancer. Overexpression of BCL6 attenuated proliferation and metastasis of GC cells in vitro and in vivo via trancriptionally repressing FZD7, a key gene of Wnt/β-catenin pathway [20]. Furthermore, BCL6 could promote the accumulation of lipid-ROS, MDA and Fe^2+^ level through FZD7/β-catenin/TP63/GPX4 pathway, which led to ferroptosis of GC cells.

In addition, our previous studies confirmed that ring finger protein 180 (RNF180) could promote the ubiquitin/proteasome pathway-dependent degradation of ras homolog gene family member C (RhoC) [21]. In this research, we found that RhoC could negatively regulate BCL6 through reducing enrichment of H3K27 acetylation on its promoter, suggesting that the low expression of BCL6 and its tumor inhibiting effect were regulated by the RNF180/RhoC pathway, which was also elucidated to significantly mediate the proliferation and metastasis of GC cells in our previous investigation [21]. In summary, we describe the mechanism by which BCL6 played a tumor inhibitory role in GC. BCL6 could be used as a prognostic biomarker and potential therapeutic target for GC patients in the future.

## Results

### Low expression of BCL6 in GC indicates more malignant clinical features and a worse prognosis

**Fig. 1:**
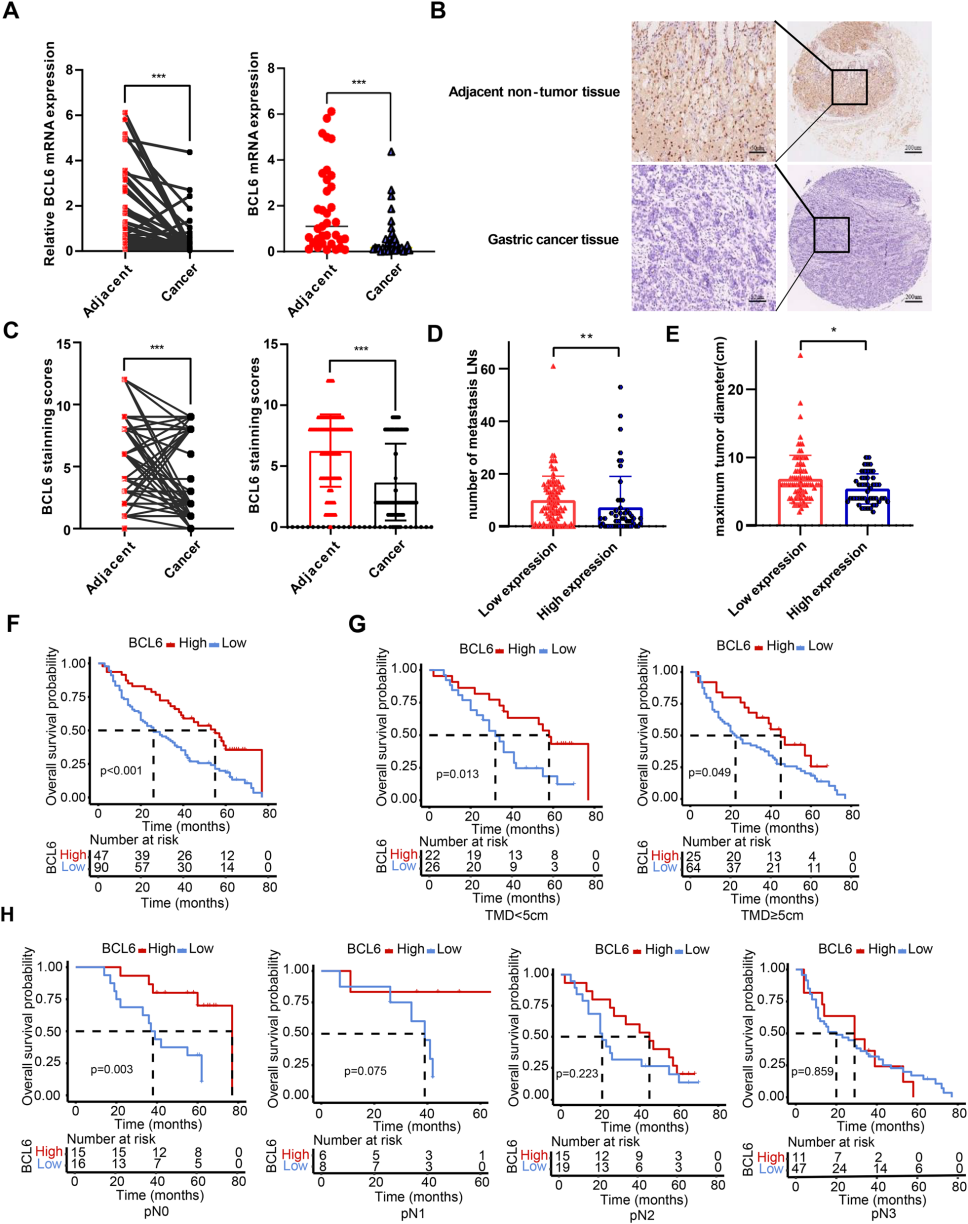
Low expression of BCL6 in GC tissues is associated with the poorer survival outcomes of GC patients. **A** The BCL6 mRNA expression in gastric cancer tissues and adjacent non-tumor tissues (***p < 0.001). **B** Immunohistochemical staining of BCL6 in gastric cancer tissues and corresponding adjacent non-tumor tissues (Original magnification, × 100 and × 400). **C** The BCL6 protein expression level in the most of gastric cancer tissues and corresponding adjacent non-tumor tissues (***p < 0.001). **D**, **E** The BCL6 protein expression level in different lymph node metastases (D) and tumor sizes (E) (*p < 0.05, **p < 0.001). **F**, **G**, **H** Keplan-Meier analysis of overall survival (F), tumor maximum diameter (TMD) (G) and lymph node node metastases subgroups (H) according to BCL6 expression level

We analyzed the mRNA expression levels of BCL6 in 36 pairs of GC tissues and adjacent non-tumor tissues using qPCR and found that the mRNA level of BCL6 in 30 cancer tissues was lower than that in the matched para-carcinoma tissues (N = 36, P < 0.001, Fig. 1A). Subsequently, we examined BCL6 protein using immunohistochemistry (IHC), which revealed that BCL6 was enriched in the cytoplasm and nucleus of gastric mucosal cells, especially in the nucleus (Fig. 1B). As shown in Fig. 1C, the immunohistochemical score showed that the level of the BCL6 protein in GC tissues was significantly lower than that in the corresponding adjacent non-tumor tissues (N = 137, P < 0.001, Fig. 1C).

The patients were then divided into low and high groups according to the median calibrated histochemical BCL6 score. The clinicopathological features of the two groups were summarized in Additional file 1: Table S1. Univariate analysis showed that compared with the high expression group of BCL6, the low expression group of BCL6 exhibited advanced pN stage (P = 0.013), increased lymph node (LN) metastasis (9.89 ± 9.12 vs. 7.16 ± 11.83, P = 0.001; Fig. 1D), and long tumor diameter (6.81 ± 3.51 vs. 5.38 ± 2.22, P = 0.012; Fig. 1E). To determine the effect of BCL6 expression on the prognosis of patients with GC, we performed a Kaplan–Meier analysis. As shown in Fig. 1F, the 5 year survival rate of GC patients with low BCL6 expression was lower than that of GC patients with high BCL6 expression (P < 0.001). Multivariate Cox regression analysis showed that BCL6 was an independent predictor of prognosis (HR 0.590, 95% CI 0.346–0.935; P = 0.026) (Table 1). We also evaluated the effects of tumor size and LN metastasis on survival outcomes by stratifying the maximum tumor diameter and pN stage. Low BCL6 expression predicted poor survival in both tumor size groups (TMD < 5 cm: P = 0.013; TM≧5 cm: P = 0.049, Fig. 1G) and pN0 subgroups (P = 0.003, Fig. 1H).

**Table 1:** Log-ranktest and multivariate Cox proportional hazard models for overall survival of gastric cancer patients

Characteristics	Case	5-YSR (%)	P-value^b^	Hazard ratio(95% CI)	P-value^c^
Gender			0.138		
Male	98	30.61			
Female	39	23.08			
Age(year)			0.145		
< 65	86	31.40			
≥ 65	51	23.53			
pT stage			0.946		
pT2	10	20.00			
pT3	9	33.33			
pT4	118	28.81			
pN stage			< 0.001	2.030 (1.084–3.800)	0.015
pN0	31	51.61			
pN1	14	50.00			
pN2	34	17.65			
pN3	58	17.24			
Tumor location			0.468		
Upper third	20	30.00			
Middle third	18	16.67			
Lower third	72	30.56			
More than 2/3 stoma	27	29.63			
Tumor size(cm)			0.082		
< 5	48	33.33			
≥ 5	89	25.84			
Lauren type			0.985		
Intestinal	32	31.25			
Diffuse	102	27.45			
Mixed	3	33.33			
Bormann type			0.516		
I	2	66.67			
II	40	22.5			
III	85	30.59			
IV	10	30.00			
BCL6 expression^a^			< 0.001	0.590 (0.346–0.935)	0.026
Low	90	21.11			
High	47	42.55			

Values in parentheses are 95 percent confidence intervals

^a^Determined by immunohistochemical staining

^b^Log-rank test

^c^Cox proportional hazards model

In conclusion, these results suggested that a decrease of BCL6 expression may promoted GC progression. Furthermore, low BCL6 expression in GC indicated larger tumor size, more LNs, and poor prognosis.

### *BCL6 impairs malignant phenotypesof GC cells in vitro*

**Fig. 2:**
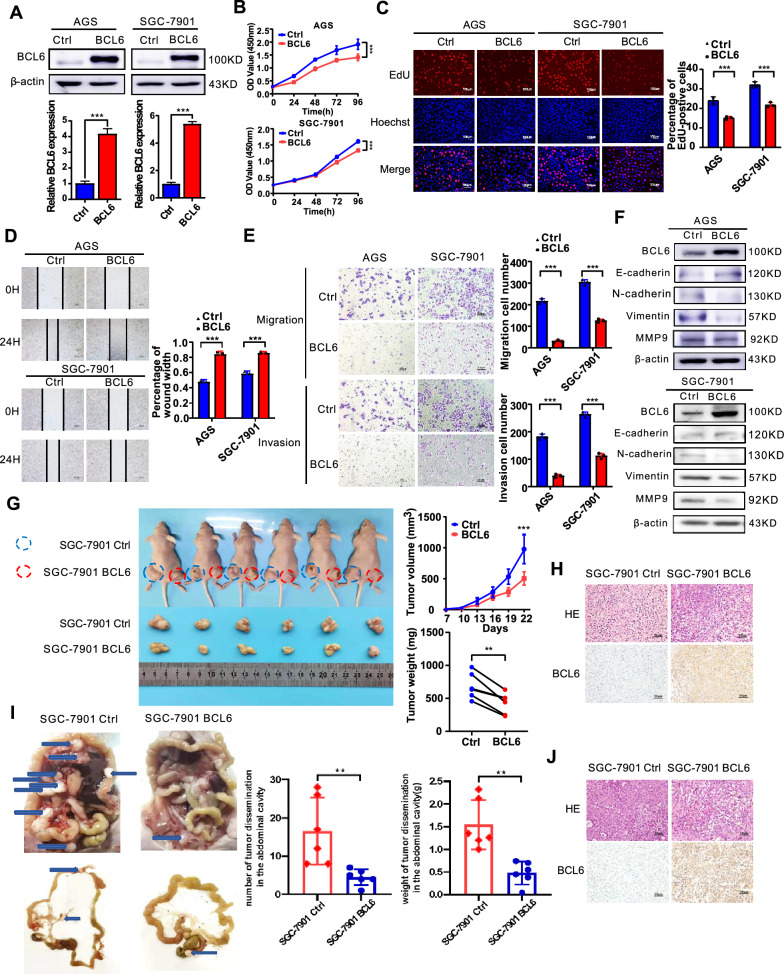
BCL6 attenuates malignant phenotypes in AGS and SGC-7901 cells. **A** Expression levels of BCL6 in AGS and SGC-7901 cells infected with BCL6 overexpression virus and empty virus were analyzed by western blotting assay (***p < 0.001). **B**, **C** BCL6 inhibits the viability and proliferation of AGS and SGC-7901 cells (***p < 0.001). **D** BCL6 inhibited the migration of AGS and SGC-7901 cells (***p < 0.001). **E** BCL6 inhibited the migration and invasion of AGS and SGC-7901 cells (***p < 0.001). **F** BCL6 inhibited the EMT process of AGS and SGC-7901 cells. **G** BCL6 inhibited the growth of SGC-7901 cells in vivo. Tumor growth curves and tumor weight shows the inhibitory effect of BCL6 on tumor in vivo (N = 6) (*p < 0.05, ***p < 0.001). **H** Representative hematoxylin and eosin (HE) staining and immunohistochemical (IHC) images of tumor xenografts derived from SGC-7901-NC, SGC-7901-BCL6 cell lines stained by BCL6 antibody (Original magnification, × 400). **I** BCL6 inhibited the intraperitoneal metastasis of SGC-7901 cells in vivo. Number and weight of disseminated tumour foci shows the metastasis inhibitory effect of BCL6 on tumor in vivo (**p < 0.01). **J** Representative HE and IHC staining of metastatic foci derived from SGC-7901-NC, SGC-7901-BCL6 cell lines (Original magnification, × 400)

To study the role of BCL6 in GC cells, we first examined the expression of BCL6 protein in seven GC cell lines and immortalized normal gastric epithelial cell (GES-1). Our results showed that the expression level of BCL6 in most GC cell lines was significantly lower than that in GES-1. The expression of AGS and SGC-7901 GC cells was at a relatively low protein expression level in these GC cell lines (Additional file 1: Fig S1A). Subsequently, we modulated BCL6 expression with BCL6 cDNA and two BCL6-specific siRNAs (Lenti-siBCL6#1 and Lenti-siBCL6#2) using lentivirus-mediated delivery into AGS and SGC-7901 cells, respectively. The cDNA-mediated BCL6 overexpression and siRNA-mediated knockdown of BCL6 in AGS and SGC-7901 cells were confirmed by western blotting (Fig. 2A, Additional file 1: Fig S1B). We found that overexpression of BCL6 inhibited the proliferation and viability of AGS and SGC-7901 cells, as shown by the CCK8, colony formation and EdU assays (Fig. 2B, C, Additional file 1: Fig S1C). In contrast, the depletion of BCL6 promoted the growth of GC cells (Additional file 1: Fig S2A–C). As mentioned earlier, the low expression of BCL6 in GC was positively correlated with LN metastasis. Therefore, we investigated the effects of BCL6 on the migration and invasion of AGS and SGC-7901 cells. In addition, the scratch and transwell assay showed that the exogenous introduction of BCL6 inhibited the migration (scratch assay: AGS cells at 24 h, P < 0.001, SGC-7901 cells at 24 h, P < 0.001, Fig. 2D; transwell migration: AGS, P < 0.001; SGC-7901, P = 0.004;). and invasion of GC cells (transwell invasion: AGS, P = 0.001, SGC-7901, P < 0.001; Fig. 2E), whereas BCL6 silencing enhanced the motility of GC cells (Additional file 1: Fig S2D, E). Therefore, we examined the expression of related indicators of the epithelial–mesenchymal transformation (EMT) process (E-cadherin, N-cadherin, vimentin, and MMP9), which are closely related to tumor metastasis. The results showed that the overexpression of BCL6 could upregulate E-cadherin and downregulate N-cadherin, vimentin, and MMP9, indicating that BCL6 could inhibit the EMT process of GC cells (Fig. 2F). The opposite result was observed in BCL6-silencing GC cells (Additional file 1: Fig S2F).

In conclusion, these results suggested that BCL6 inhibited the proliferation, invasion, and migration of GC cells.

### *BCL6 inhibits the growth and intraperitoneal dissemination of GC cells *in vivo

We subcutaneously injected BCL6-overexpressing or control AGS and SGC-7901 GC cells into the flanks of nude mice, and tumor growth was closely observed over 22 days. The growth rate of BCL6-overexpressing SGC-7901 and AGS cells was significantly lower than that of control cells (Fig. 2G, Additional file 1: Fig S3). Simultaneously, the weight of the harvested tumor mass showed that BCL6 inhibited the proliferation of SGC-7901 and AGS cells in nude mice (Fig. 2G). Tumors from nude mice were sectioned and subjected to hematoxylin and eosin (HE) staining and IHC analysis. The results showed that the staining intensity of BCL6 in the tumor tissue of the BCL6-overexpressing group was significantly stronger than that in the control group (Fig. 2H). Subsequently, we constructed an intraperitoneal dissemination tumor model to explore the effect of BCL6 on the metastasis of GC cells in nude mice. The results showed that compared with the control group, the tumor metastases in the abdominal cavity of nude mice in the BCL6 overexpression group were substantially reduced, and the weight of the corresponding metastases was also significantly reduced (Fig. 2I). Tumor metastases from nude mice were sectioned and subjected to HE and IHC staining (Fig. 2J).

### BCL6 suppresses the activation of theWnt/β-catenin pathway by transcriptionally repressing FZD7 in GC cells

**Fig. 3:**
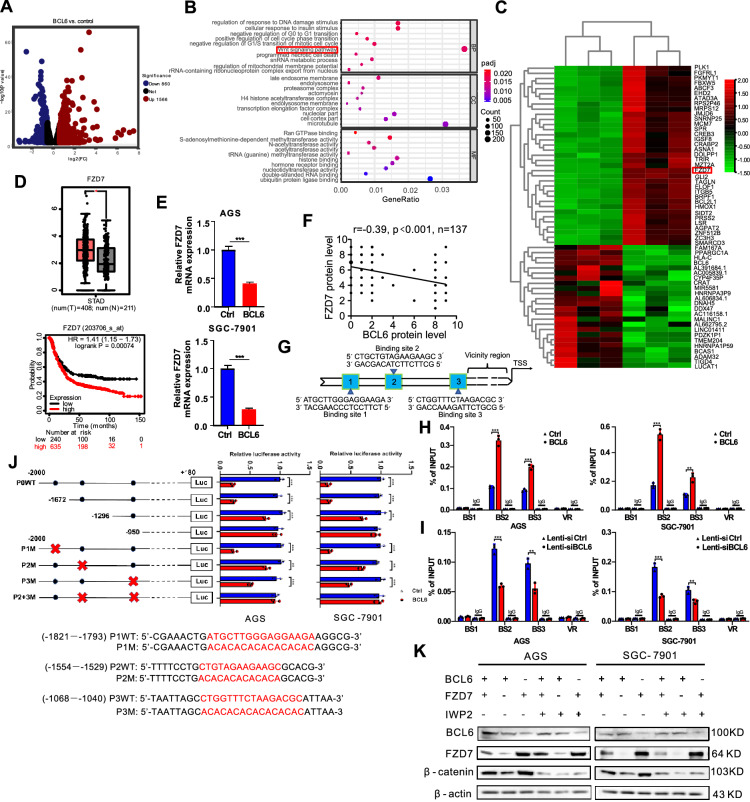
BCL6 suppresses Wnt/β-catenin signaling pathway via directly transcriptionally repressing FZD7. **A** Volcano plots showed differentially expressed genes (DEGs) (Fold change > 1.5). **B** Gene ontology (GO) enrichment analysis of BCL6 downstream genes. Wnt signaling pathway was shown to be enriched significantly. **C** Clustering and heatmap analysis of differential gene expression in the control group and the BCL6 overexpression group in AGS cell. **D** GEPIA database analysis showed that the expression levels of FZD7 in GC tissues were significantly higher than adjacent non-tumor tissues; KM-plotter database analysis showed that gastric cancer patients with high expression of FZD7 had a worse prognosis (*p < 0.05). **E** BCL6 could repress FZD7 expression at the transcriptional level in GC cells (***p < 0.001). **F** Correlations between the immunohistochemical expression of FZD7 and BCL6 in human GC tissues. FZD7 negatively correlates with BCL6 in human GC tissues (N = 137). **G** Schematic diagram of the FZD7 promoter region and the putative BCL6 binding sites. **H**, **I** ChIP analysis of BCL6 binding to the FZD7 promoter in the AGS and SGC-7901 cells. Different regions of the FZD7 promoter contain different putative BCL6 binding sites are shown in **G**. The matched IgG was used as a negative control and vicinity region (VR) (− 901–0 bp) also used as a control (**p < 0.01, ***p < 0.001). **J** Luciferase activities of FZD7 promoter reporter vectors in AGS and SGC-7901 cells. Red letters in each binding region indicate the putative or mutated BCL6 binding sequences (**p < 0.01, ***p < 0.001). **K** β-catenin protein level of GC cells after BCL6 or FZD7 overexpression combined with Wnt inhibitor IWP2 treatment (5 µM, 12 h)

BCL6 is a transcriptional repressor. Immunohistochemical studies revealed that BCL6 was highly expressed in the nuclei of gastric mucosal cells. Thus, we speculated that this protein could regulate gene expression in GC cells. Therefore, we performed transcriptome sequencing to analyze the mRNA profiles in the control group and BCL6-overexpressing AGS cells, which indicated that the expression of 2426 genes (DEGs) changed significantly overall (FC > 1.5) (Fig. 3A). GO analysis of the sequencing results showed that the Wnt signaling pathway was enriched in BCL6 downstream genes (Fig. 3B). Meanwhile, FZD7, the key receptor for Wnt/β-catenin has attracted our attention (FC > 2) (Fig. 3C). The expression of FZD7 in patients with GC and their impact on prognosis were analyzed online using the Kaplan–Meier Plotter (KM-Plotter) database (http://kmplot.com/analysis/). The results showed that FZD7 was highly expressed in patients with GC and predicted poor prognosis (Fig. 3D). The expression of FZD7 was further verified using qPCR in the control group and BCL6-overexpressing AGS and SGC-7901 cells (Fig. 3E). We further investigated β-catenin, the key molecule of Wnt/β-catenin pathway, after treatment with a Wnt inhibitor, the upregulation of β-catenin caused by BCL6 knockdown was completely abolished in the two GC cell lines (Additional file 1: Fig S4A).

As the receptor of Wnt, FZD7 is responsible for transmitting extracellular Wnt signals intracellularly, thus affecting the expression level of β-catenin [22]. Therefore, we speculated that BCL6 might inhibit the intracellular transmission of Wnt by inhibiting FZD7. The correlation between BCL6 and FZD7 expression in GC tissues was further analyzed using human GC tissue microarrays. The results indicated that FZD7 was negatively correlated with BCL6 in GC tissues (Fig. 3F, Additional file 1: Fig S4B). IHC also showed that the staining intensity of FZD7 in the tumor tissue of BCL6-overexpressing nude mice was significantly weaker than that in the control group (Additional file 1: Fig S4C). BCL6 is a classic transcriptional suppressor; therefore, we speculated that BCL6 was a direct transcriptional repressor of FZD7, which was verified through ChIP and luciferase reporter gene assays. Several putative BCL6-binding sites in the promoter of FZD7 were predicted by the JASPAR database (Fig. 3G). Chromatin immunoprecipitation (ChIP) analysis showed that BCL6 was recruited to promoter regions containing binding sites 2 and 3 (Fig. 3H). The results showed that BCL6-overexpressing GC cells had significantly more enrichment of BCL6 on the BS2 and BS3 of FZD7 promoter region compared with the control group. While the enrichment of BCL6 on the BS2 and BS3 of FZD7 promoter region in BCL6-knockdown GC cells was significantly reduced. Similar results were obtained in 293 T cells (Fig. 3I, Additional file 1: Fig S4D, E). Truncated FZD7 promoters or site-directed mutagenesis combined with luciferase reporter assays indicated that these 2 binding sites in the FZD7 promoter mediated the repression of promoter activity induced by BCL6 (Fig. 3J). Compared with GC cells infected only with the BCL6 overexpression lentivirus group, the β-catenin protein level in the BCL6 overexpression combined with the FZD7 overexpression group was significantly restored. However, the β-catenin protein level decreased significantly after the Wnt inhibitor IWP-2 was incubated based on the overexpression (Fig. 3K).

### BCL6 undermines viability and motility of GC cell by suppressing FZD7

**Fig. 4:**
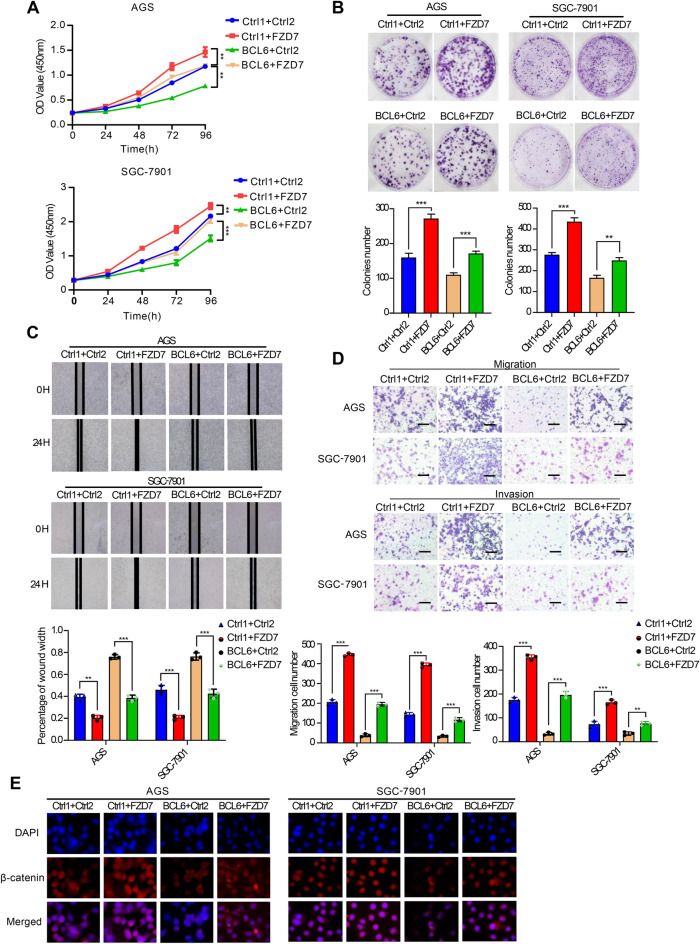
BCL6 exerts its tumor suppressor function by repressing FZD7. **A** CCK8 assay showed that FZD7 overexpression reversed BCL6-inhibited growth in AGS and SGC-7901 cells (**p < 0.01, ***p < 0.001). **B** Colony formation assay showed that FZD7 overexpression reversed BCL6-inhibited colony formation in AGS and SGC-7901 cells (**p < 0.01, ***p < 0.001). **C**, **D** FZD7 overexpression attenuated the inhibition of BCL6 on cell invasion and migration, as measured by wound healing and transwell assay (Scale bar, 100 μm) (**p < 0.01, ***p < 0.001). **E** Immunofluorescence staining for β-catenin (red) and 4′,6-diamidino-2-phenylindole (DAPI) (blue) in AGS and SGC-7901 cells transfected with control vector (Ctrl1 + Ctrl2, Ctrl1 + FZD7, BCL6 + Ctrl2) or co-transfected BCL6 plasmid and FZD7 plasmid (BCL6 + FZD7) (Original magnification, × 400)

Functional rescue experiments showed that the overexpression of FZD7 significantly reversed the inhibitory effect of BCL6 on proliferation, invasion, and migration of GC cells (Fig. 4A–D). In addition, immunofluorescence staining was performed to determine the β-catenin levels in AGS and SGC-7901 cells. The results showed that nuclear β-catenin levels were reduced after BCL6 overexpression and were restored when FZD7 was simultaneously overexpressed (Fig. 4E). WB analysis of cytoplasmic (C) and nuclear (N) extracts was performed. These results also verified this conclusion (Additional file 1: Fig S4F). Therefore, BCL6 could directly repressed the transcription of FZD7 to inhibit the Wnt/β-catenin signaling pathway and acted as a tumor suppressor in GC.

### BCL6 facilitates ferroptosis of GC cells by suppressing FZD7/β-catenin/TP63/GPX4 pathway

**Fig. 5:**
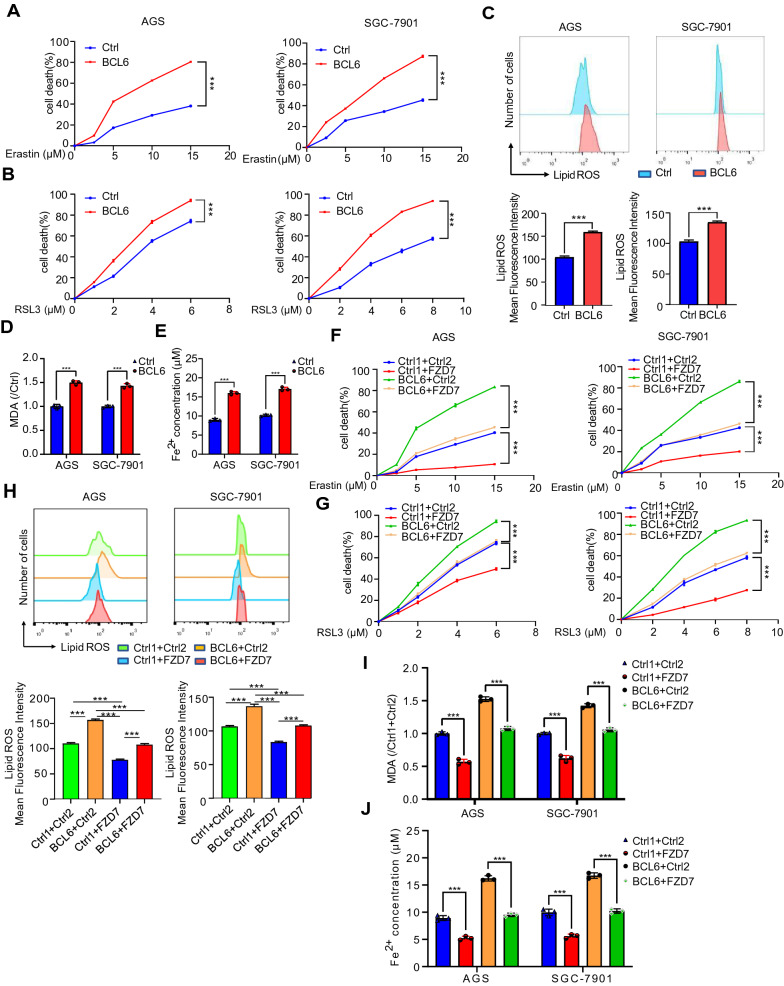
FZD7 attenuates the BCL6-induced ferroptosis facilitation of GC cells. **A**, **B** AGS and SGC-7901 cells were transfected with control vector or BCL6 plasmid. After 6 h, cells were seeded in 96-well plates overnight. Cell death was detected by MTT assay for cells treated with or without corresponding concentration of erastin and RSL3 after 48 h (***p < 0.001). **C**, **D**, **E** Lipid peroxidation (Lipid ROS), lipid Oxidation (MDA) and Fe^2+^ level were measured in AGS and SGC-7901 cells transfected with control vector or BCL6 plasmid (***p < 0.001). **F**, **G** AGS and SGC-7901 cells were transfected with control vector (Ctrl1 + Ctrl2, Ctrl1 + FZD7, BCL6 + Ctrl2) or co-transfected BCL6 plasmid and FZD7 plasmid (BCL6 + FZD7). Then, cells were treated as in **A**, **B**, and cell death was detected (***p < 0.001). **H**, **I**, **J** Lipid ROS, MDA and Fe^2+^ level were measured in AGS and SGC-7901 cells transfected with control vector (Ctrl1 + Ctrl2, Ctrl1 + FZD7, BCL6 + Ctrl2) or co-transfected BCL6 plasmid and FZD7 plasmid (BCL6 + FZD7) (***p < 0.001)

Recent studies have shown that FZD7 could promote ferroptosis through β-catenin-TP63-GPX4 pathway in ovarian cancer [23]. We have confirmed that FZD7 was the direct target of BCL6 in GC. Therefore, we tried to explore whether BCL6 could affect the occurrence of ferroptosis in GC cells. First of all, we found that the overexpressed BCL6 increased ferroptosis inducer RSL3- or erastin-induced cell death, indicating that BCL6 could promote the occurrence of ferroptosis in GC cells (Fig. 5A, B). Then, we detected the level of lipid peroxidation (lipid-ROS), lipid oxidation, MDA and Fe^2+^ concentration, which are the key substance leading to ferroptosis [24]. The results showed that BCL6 could promote the up-regulation of lipid-ROS, MDA and Fe^2+^ level in GC cells (Fig. 5C–E).

**Fig. 6:**
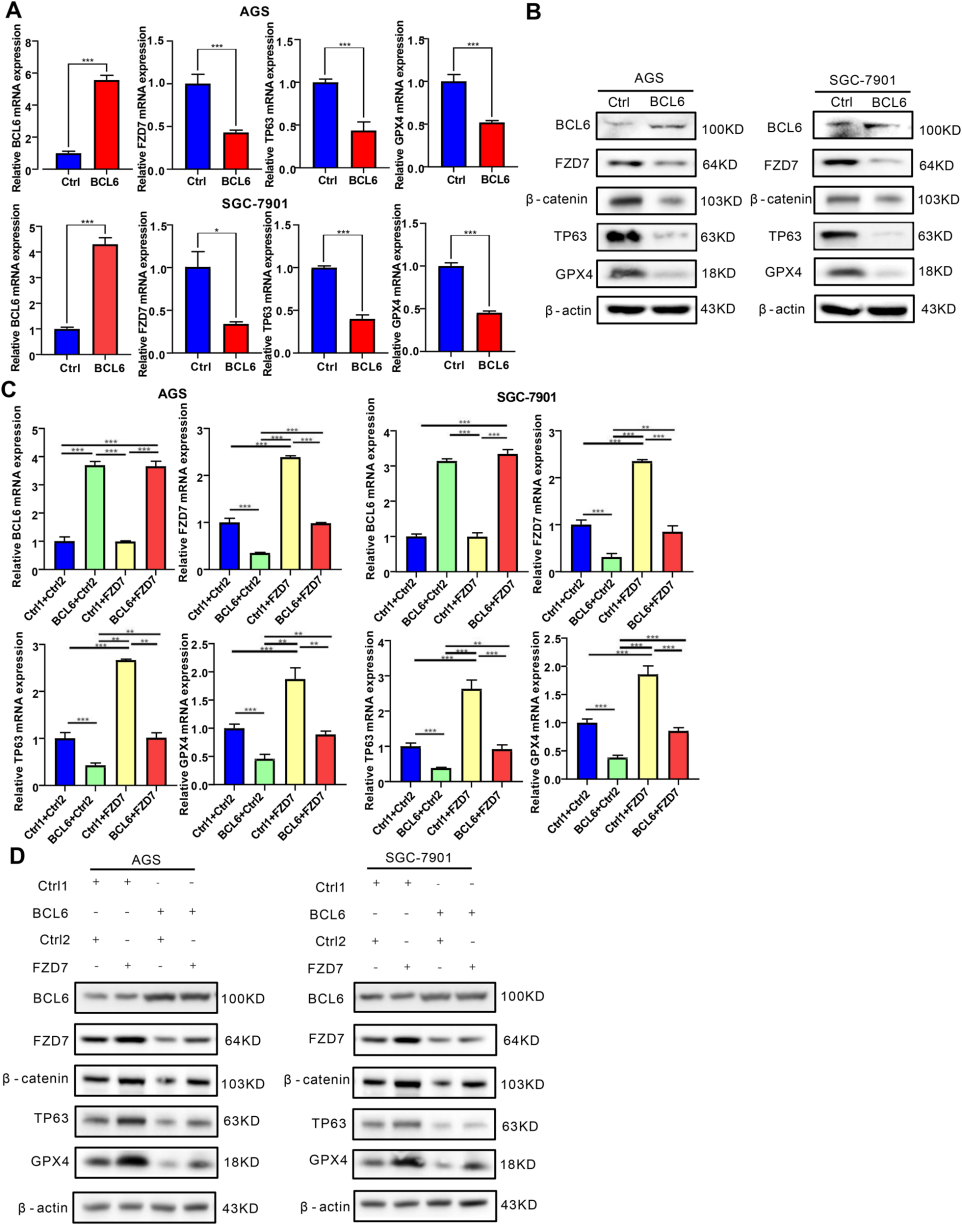
BCL6 promotes ferroptosis of GC cells through FZD7/β-catenin/TP63-GPX4 pathway. **A** BCL6, FZD7, TP63, GPX4 mRNA levels in GC cells transfected with control vector or BCL6 plasmid (*P < 0.05, **p < 0.01, ***p < 0.001). **B** Western blot for BCL6, FZD7, β-catenin, TP63, GPX4 and β-actin in AGS and SGC-7901 cells transfected with control vector or BCL6 plasmid. **C** BCL6, FZD7, TP63, GPX4 mRNA levels in AGS and SGC-7901 cells transfected with control vector (Ctrl1 + Ctrl2, Ctrl1 + FZD7, BCL6 + Ctrl2) or co-transfected BCL6 plasmid and FZD7 plasmid (BCL6 + FZD7) (**p < 0.01, ***p < 0.001). **D** Western blot for BCL6, FZD7, β-catenin, TP63, GPX4 and β-actin in AGS and SGC-7901 cells treated as in **C**

In order to further clarify whether BCL6 promoted ferroptosis in GC cells through FZD7, we overexpressed BCL6 and FZD7 in GC cells at the same time, and then determined cell death. We found that FZD7 could reverse the facilitation of BCL6 on erastin or RSL3- induced cell death (Fig. 5F, G). In addition, we detected lipid-ROS, MDA and Fe^2+^ concentration, and found that FZD7 could reverse the promoting effect of BCL6 on lipid-ROS accumulation, MDA and Fe^2+^ level (Fig. 5H–J). In short, FZD7 could reverse the occurrence of ferroptosis induced by BCL6. From a mechanistic standpoint, we detected the expression of TP63 and ferroptosis marker GPX4. The results showed that BCL6 could significantly repress the expression of TP63 and GPX4 in GC cells (Fig. 6A, B). Moreover, β-catenin, TP63 and GPX4 levels could be restored by FZD7 in BCL6 overexpressed GC cells (Fig. 6C, D). Then, we explored the role of TP63 in this process, and found that depletion of TP63 could reverse the promoting effect of FZD7 on GPX4, while overexpression of TP63 could restore the inhibitory effect of BCL6 on GPX4. In addition, we also found that TP63 depletion could in turn reduce the expression of FZD7 (Additional file 1: Fig S5A, B). In general, we found that BCL6 promoted ferroptosis in GC cells on the FZD7-β-catenin-TP63-GPX4 pathway.

### The expression and role of BCL6 in GC cells are strengthened by the RNF180/RhoC pathway

**Fig. 7:**
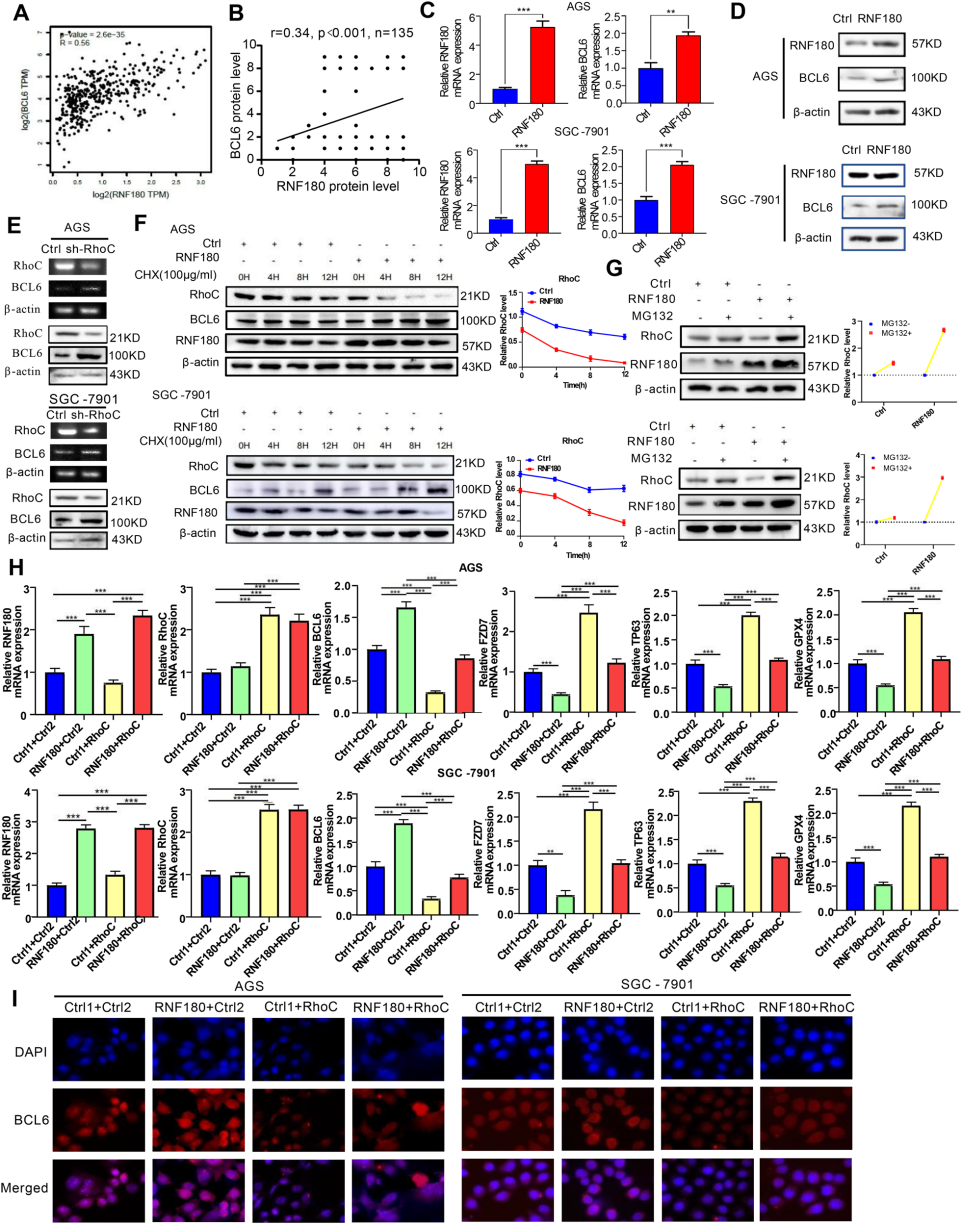
Expression of BCL6 in GC cells is mediated by RNF180/RhoC pathway. **A** Scatter plot shows the positive correlation between RNF180 and BCL6 mRNA expression levels in STAD tumor (N = 408), according to the online GEPIA database; **B** and the IHC of TMAs (N = 135); **C**, **D** Expression levels of BCL6 in AGS and SGC-7901 cells transfected with RNF180 plasmid and control vector were analyzed by qPCR and western blot (**p < 0.01, ***p < 0.001). **E** Expression levels of BCL6 in AGS and SGC-7901 cells transfected with short hairpin RNA (shRNA) for RhoC or control vector were analyzed by qPCR and western blot. **F** AGS and SGC-7901 cells were transfected RNF180 plasmid or control vector. After 48 h, cells were treated with 100 mg/ml CHX at the indicated time point. The RhoC protein was measured by western blot. **G** AGS and SGC-7901 cells were transfected RNF180 plasmid or control vector. After 48 h, cells were incubated with 10 μM MG132 for 24 h.The RhoC protein was measured by western blotting. **H** RNF180, RhoC, BCL6, FZD7, TP63 and GPX4 mRNA expression in AGS and SGC-7901 cells transfected with control vector (Ctrl1 + Ctrl2, Ctrl1 + RhoC, RNF180 + Ctrl2) or co-transfected RNF180 plasmid and RhoC plasmid (RNF180 + RhoC) (***p < 0.001). **I** Immunofluorescence staining for BCL6 (red) and 4′,6-diamidino-2-phenylindole (DAPI) (blue) in AGS and SGC-7901 cells transfected with control vector (Ctrl1 + Ctrl2, Ctrl1 + RhoC, RNF180 + Ctrl2) or co-transfected RNF180 plasmid and RhoC plasmid RNF180 + RhoC) (Original magnification, × 400)

Our previous studies showed that RNF180, a tumor suppressor in GC, inhibited the proliferation and movement of GC cells [25]. Gene Expression Profiling Interactive Analysis (GEPIA) database (http://gepia.cancer-pku.cn/) analysis showed that there was a strong positive correlation between BCL6 and RNF180 expression (Fig. 7A). And our IHC results also demonstrated that BCL6 expression was positively correlated with RNF180 in the GC tissue microarrays (Fig. 7B, Additional file 1: Fig S6A). Therefore, we investigated whether RNF180 regulated the expression of BCL6. RNF180 was overexpressed in AGS and SGC-7901 GC cells to verify whether it affected the expression level of BCL6. The results showed that RNF180 increased the transcription and protein levels of BCL6 in GC cells (Fig. 7C, D).

RNF180 is an E3 ubiquitin ligase that directly promotes ubiquitination and subsequent degradation of its target gene at the protein level but cannot directly promote the expression of its downstream genes at the transcriptional level [26]. Therefore, RNF180 indirectly regulated BCL6 by forming a regulatory axis with a specific molecule. Our previous studies showed that RNF180 could promote proteasome-pathway-dependent degradation of the oncogene RhoC in GC cells [21]. Therefore, we investigated whether RNF180 promoted the expression of BCL6 through RhoC. First, RhoC was knocked down in AGS and SGC-7901 GC cells, and the expression of BCL6 was determined using western blotting and qPCR. The results showed that the mRNA and protein levels of BCL6 were significantly increased (Fig. 7E). IHC results also suggested a negative correlation between RhoC and BCL6 levels in GC tissues (Additional file 1: Fig S6B, C). Mechanically, it has been reported that RhoC could promote the expression of HDAC1 in GC [27]. This was also confirmed in our study (Additional file 1: Fig S7A). It is well known that HDAC1-mediated histone deacetylation has obvious inhibitory effect on the transcription of multiple genes [28, 29]. We found that RhoC could significantly reduce the enrichment of ac-H3K27 on the promoter region of BCL6 by promoting the expression of HDAC1, and thus exert transcriptional repression on BCL6 (Additional file 1: Fig S7B–E). Therefore, we speculated that RNF180 might reversed the decreased expression of BCL6 in GC cells by promoting the degradation of RhoC through the proteasome pathway.

Then, Cycloheximide (CHX) was incubated in control and RNF180-overexpressing AGS and SGC-7901 GC cells, and the protein levels of RhoC were examined at fixed time intervals. The results showed that RNF180 promoted the degradation of RhoC (Fig. 7F). MG132 (10 µM) was added to both cell lines. The results revealed that with the inhibition of the proteasome, RNF180 was unable to promote the proteasome-dependent degradation of RhoC; thus, the levels of RNF180 and RhoC protein were accumulated (Fig. 7G). To further determine the dependence of RhoC on the increase in BCL6 caused by RNF180, we upregulated RhoC in AGS and SGC-7901 cells overexpressing RNF180. The results showed that overexpression of RhoC reversed the upregulation of BCL6 and downregulation of its downstream genes induced by RNF180 at the mRNA and protein levels (Fig. 7H, Additional file 1: Fig S8A). In addition, immunofluorescence staining was performed to determine the BCL6 levels in AGS and SGC-7901 cells. We found that nuclear BCL6 levels were upregulated after RNF180 overexpression and were reversed when RhoC was simultaneously overexpressed (Fig. 7I). WB analysis of cytoplasmic (C) and nuclear (N) extracts was performed. These results equally verified this conclusion (Additional file 1: Fig S8B). Similarly, BCL6 depletion restored the downregulation of FZD7 and its downstream genes induced by RNF180 overexpression or RhoC depletion (Additional file 1: Fig S9A, B; S10A, B).

**Fig. 8:**
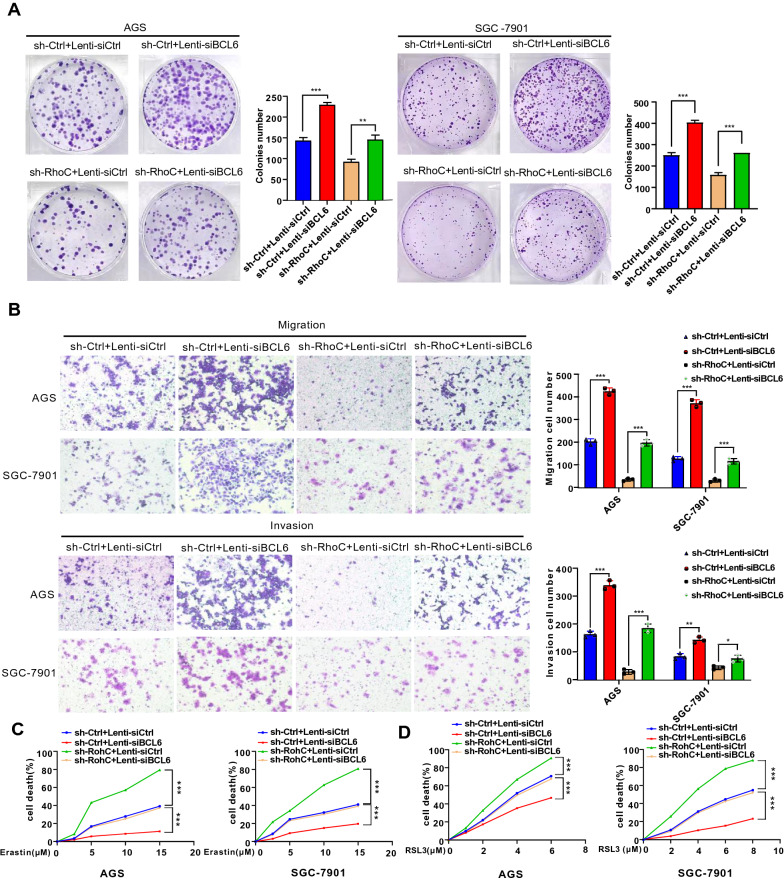
Malignancy reppression and ferroptosis facilitation of BCL6 in GC cells are down-regulated by RhoC. **A**, **B** BCL6 depletion reversed the inhibition of cell proliferation, migration and invasion induced by RhoC depletion in AGS and SGC-7901 cells (Scale bar, 100 μm) (*p < 0.05, **p < 0.01, ***p < 0.001). **C**, **D** BCL6 depletion reversed cell death induced by erastin or RSL3 in RhoC-depleted AGS and SGC-7901 cells (***p < 0.001)

The the colony formation and transwell assays revealed that depletion of BCL6 could reverse the inhibitory effect of RhoC depletion or RNF180 overexpression on the proliferation, migration and invasion capacities, respectively, of the two GC cell lines (Fig. 8A, B, Additional file 1: Fig S11A, B). Meanwhile, we found that BCL6 depletion could reverse the promotion of RhoC depletion or RNF180 overexpression on erastin or RSL3- induced cell death (Fig. 8C, D, Additional file 1: Fig S11C, D). In short, the malignancy inhibition and ferroptosis facilitation of BCL6 mediated FZD7 repression could be strengthened by RNF180/RhoC pathway in GC cells.

## Discussion

BCL6 is a transcriptional repressor that is closely related to transcriptional regulation, epigenetics, tumorigenesis and progression [2, 30, 31]. BCL6 may have a specific role in tumor progression, depending on the tissue and organ involved. For example, BCL6 played a tumor-promoting role in breast cancer and most lymphomas, whereas it could act as a tumor suppressor in medulloblastoma and HB-EGF-CTF-positive GC [3, 6–8]. However, its role in other GC has not yet been fully elucidated. In this study, we provided the clinical and experimental evidence supporting the inhibitory effect of BCL6 on GC. Clinicopathological data analysis of GC patients from our hospital indicated that the low expression of BCL6 was closely associated with later pN stage, larger tumor size, and poor survival outcomes. Further analysis showed that the low BCL6 expression in patients with GC was an independent risk factor. Similarly, this conclusion was indirectly confirmed in cell function and animal experiments: BCL6 significantly inhibited the proliferation and metastasis of GC cells. Our findings suggested that BCL6 might be a potential biomarker for predicting the prognosis of patients with GC, and these data provided a basis for further investigation into whether BCL6 might serve as a novel target for gastric carcinogenesis and metastasis.

Then, we screened potential downstream direct target genes of BCL6 in GC cells using transcriptome sequencing. Considering that the Wnt signaling pathway was significantly enriched by GO analysis of the sequencing results, we further considered FZD7 as a BCL6 potential direct target gene, which was a key gene in the Wnt signaling pathway [32]. We performed a simple validation in both cell lines and found that BCL6 could indeed repress FZD7. As a transmembrane receptor, FZD7 could influence Wnt signaling and effectively inhibit the degradation of β-catenin through the phosphorylation of downstream protein kinases [33, 34]. Accumulated β-catenin entered the nucleus and bound to the TCF/LEF transcription factor family, which could promote the transcription of target genes and played a pro-oncogenic role [35–37]. FZD7 was closely related to advanced GC and the poorer prognosis of GC patients. Additionally, FZD7 effectively promoted the proliferation, migration, and invasion of GC cell [33]. This study suggested that BCL6 could directly bind to the promoter of FZD7 and repress its transcription. The addition of Wnt inhibitors and rescue experiments further clarified that BCL6 inhibits the Wnt/β-catenin signaling pathway through direct transcriptional repression of the oncogene FZD7, thereby exerting its tumor suppressor effect in GC.

Ferroptosis, as a new way of programmed cell death, has been paid more and more attention in tumor-related research in recent years, mainly characterized by the accumulation of lipid peroxidation products, which will lead to cell death after reaching lethal dose [10, 11]. GPX4-GSH defense system is a classic way to prevent ferroptosis, in which GPX4 can eliminate lipid peroxidation products. When its expression is down-regulated or its activity decreases, lipid peroxidation products are greatly accumulated and promote the occurrence of ferroptosis [18, 19]. Recent studies have shown that FZD7 could inhibit ferroptosis in ovarian cancer through β-catenin/TP63/GPX4 pathway [23]. Therefore, we investigated for the first time whether BCL6 could regulate the occurrence of ferroptosis. Our research showed that BCL6 could promote the occurrence of ferroptosis in GC cells and identified the FZD7/β-catenin/TP63/GPX4 pathway that played a role in this process.

Furthermore, we found a positive correlation between the expression of RNF180 and BCL6 using IHC staining of GC tissues in our study. Our previous study showed that RNF180, a suppressor gene that inhibits GC growth and LN metastasis, could be an independent prognostic indicator for GC [25]. RNF180, an E3 ubiquitin ligase, belongs to the ubiquitin–proteasome system, plays an essential role in post-translational modification, and is closely related to the process of tumorigenesis and progression [38, 39]. Our previous study also showed that RNF180 could directly promote proteasome-mediated degradation of the oncogene RhoC [21]. In this study, we found that RhoC could negatively regulate BCL6 through decreasing the enrichment of H3K27 acetylation on its promoter mediated by HDAC1, and IHC staining of tissue microarray also showed that RhoC was significantly negatively correlated with BCL6 expression. Furthermore, BCL6 depletion induced malignancy promotion and ferroptosis resistance could be weaken by RhoC depletion or RNF180 overexpression in GC. In conclusion, BCL6 expression and its tumor suppressor role in GC could be strengthened by RNF180/RhoC pathway.

## Conclusions

**Fig. 9:**
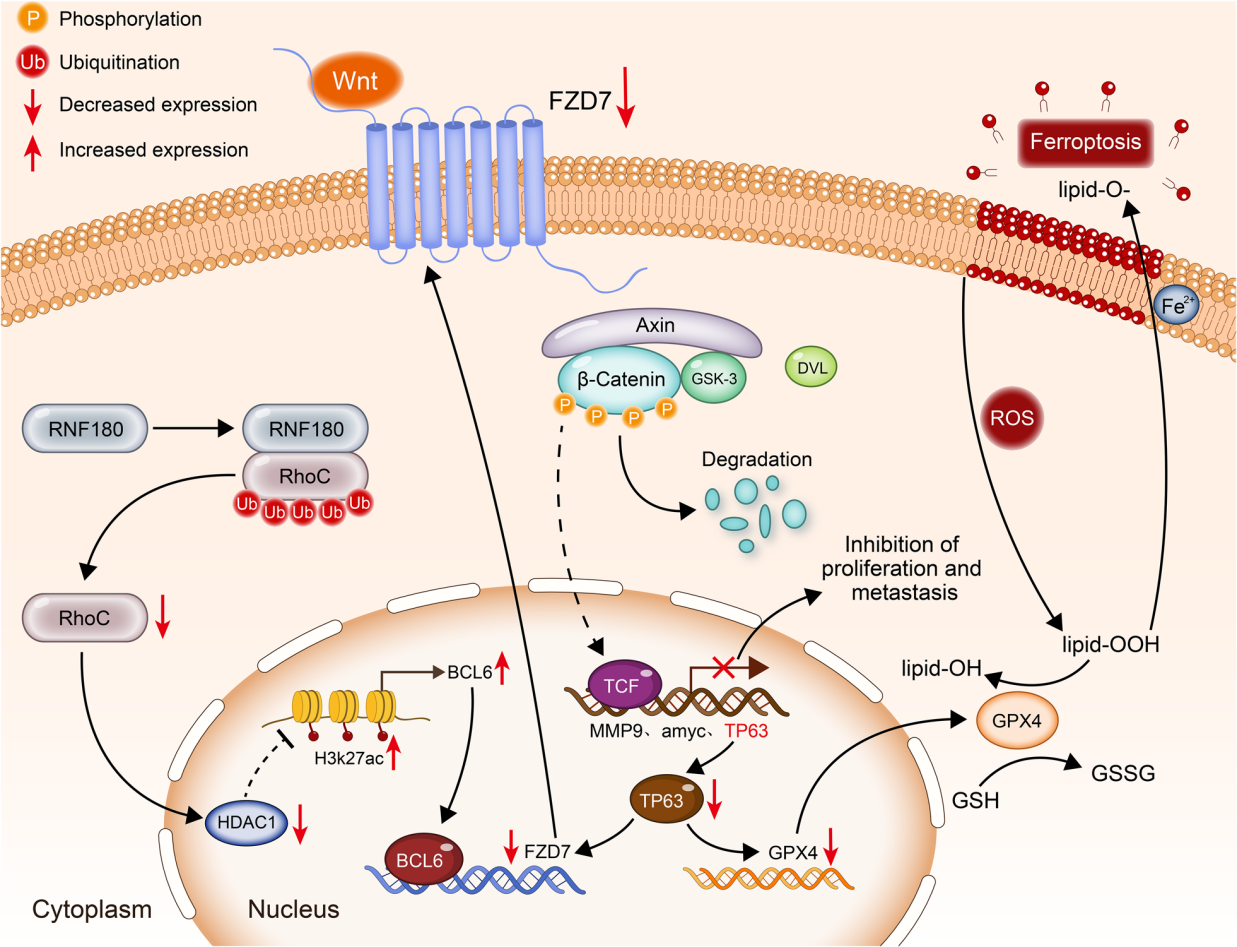
Schematic diagram. RNF180 increased the ubiquitin–proteasome pathway dependent degradation of RhoC. Reduced RhoC could further promote the H3K27ac enrichment on BCL6 promoter through the decrease of HDAC1, thus promoting the expression of BCL6. Then, accumlated BCL6 repressed FZD7 to inhibit Wnt/β-catenin signaling pathway and undermined the proliferation and metastasis of GC cells, meanwhile, down-regulated FZD7 induced ferroptosis through further repressed GPX4 mediated by decreased TP63

In summary, we conducted a systematic and profound study on the specific role of BCL6 in GC. BCL6 could transcriptionally repress the Wnt signaling receptor FZD7 and further repress the Wnt/β-catenin signaling pathway to inhibit the malignant progression of GC cells. And BCL6 could also promote ferroptosis in GC cells through FZD7/β-catenin/TP63/GPX4 pathway. In addition, the expression and function of BCL6 in gastric cancer could be strengthened by the RNF180/RhoC pathway (Fig. 9). From a clinical perspective, low BCL6 expression was closely related to larger tumor size, later pN stage, and poor survival outcome, which might serve as a compelling candidate biomarker for predicting metastasis and prognosis in GC patients.

## Materials and methods

### Tissue sample collection and follow-up

Gastric cancer and adjacent non-tumor tissues were collected from 137 GC patients who underwent radical gastrectomy at the Tianjin Medical University Cancer Hospital (Tianjin, China), Renji Hospital of Shanghai Jiao Tong University School of Medicine (Shanghai, China), and Xijing Hospital of Air Force Medical University (Xi’an, China) between January 2004 and December 2007. Upon completion of the surgery, postoperative follow-up examinations were conducted every 3–6 months and completed in September 2012. The median survival was 34.0 months (range: 2–75 months). In addition, 36 pairs of GC and adjacent non-tumor tissues were randomly selected for RNA extraction from October 2019 to January 2020. None of the selected patients had received neoadjuvant therapy prior to gastrectomy. All patients provided formal written consent to use their tissues and clinical data in this study. Ethics committee permission and study protocols were approved by the Institutional Research Ethics Committee of the Cancer Institute of Tianjin Medical University and the hospital (Tianjin, China).

### Immunohistochemistry (IHC)

The expression of RNF180, RhoC, BCL6 and FZD7 in 143 pairs of tumor and adjacent non-tumor tissues was examined using IHC. Rabbit anti-RNF180 antibody (1:150) (GTX119301, GeneTex), mouse anti-RhoC (1:50) (sc-393090, Santa Cruz Biotechnology), Rabbit anti-BCL6 antibody (1:200) (Cell Signaling Technology) and mouse anti-FZD7 antibody (Santa Cruz Biotechnology) were used as the primary antibodies. The positive cell rate and staining intensity were scored. IHC was performed as previously described [40]. IHC staining was evaluated by two blinded pathologists, and discrepant scores were re-examined to reach a consensus. The expression levels of RNF180, RhoC, BCL6 and FZD7 were evaluated by multiplying the staining intensity and frequency scores. Staining intensity was graded as follows: negative, 0; weak, 1; medium, 2; and strong, 3. The staining frequency score was classified as: 0–25%, 1; 26–50%, 2; 51–75%, 3; and 76–100%, 4.

### RNA sequencing and analysis

AGS GC cells were stably infected with BCL6 overexpression and control lentiviruses. Total RNA was extracted using the TRIzol reagent (Invitrogen). RNA integrity was assessed using an Agilent 2100 Bioanalyzer (Agilent Technologies, Santa Clara, California, USA). cDNA libraries were constructed according to the manufacturer’s instructions (TruSeq Stranded mRNA LT Sample Prep Kit). Differentially expressed genes (DEG) were analyzed using hierarchical cluster analysis to explore gene expression patterns. DEGs were enriched by GO using the R software. Transcriptome sequencing and analysis were performed by Nuohe Zhiyuan Biotechnology (Beijing, China).

### Chromatin immunoprecipitation (ChIP) analysis

For ChIP assays, 5 × 10^7^ AGS, SGC-7901 and 293 T cells stably overexpressing or depleting BCL6 and control lentivirus were prepared using a ChIP assay kit (Cell Signaling Technology) according to the manufacturer’s instructions. A 10 μL sample of the diluted chromatin sample was used as the input sample. In addition, equal volumes (2 μL) of anti-BCL6 and normal rabbit IgG antibodies were added to the samples for IP analysis. After DNA purification, the immunoprecipitated DNA was sequenced using RT-qPCR. Amplification efficiency was calculated as enrichment relative to the input. The primers used for ChIP-qPCR are listed in Additional file 1: Table S2.

### Luciferase assay

Luciferase reporter assays were performed as previously described. Briefly, the cells were seeded in a 24-well plate. AGS GC cells stably overexpressing BCL6 and control lentivirus were transfected with 1 μg truncated FZD7 promoter-luciferase reporter, 0.5 μg empty vector, and 0.1 μg β-galactosidase reporter. After 48 h of transfection, cells were harvested in lysis buffer. Luciferase activity was measured using a dual-luciferase reporting analysis system (Promega, Madison, WI, USA) according to the manufacturer’s instructions on a Varioscan flash instrument (Thermo Fisher Scientific, Carlsbad, California, USA).

### Lipid peroxidation assays

C11-BODIPY581/591 (Thermo Fisher Scientific, Waltham, MA, USA) was used to detect the level of lipid peroxidation. Cells were incubated in RPMI-1640 or F12 medium with C11-BODIPY581/591 (1 μM) for 30 min at 37 °C. Flow cytometry was carried out at the absorbance of 488/510 nm.

### Measurement of MDA

According to the manufacturer’s instructions, the level of malondialdehyde (MDA) was determined by using the Lipid Peroxidation MDA Assay Kit (Beyotime Institute of Biotechnology). Cells were lysed by 150 mL MDA lysis buffer on ice, followed by centrifugation at 12,000 × g for 10 min under 4 °C. 100 mL of lysate supernatant was mixed with 200 mL of malondialdehyde solution and incubated for 15 min at 100 °C under light-proof conditions. The mixtures was then centrifuged at 1000 g for 10 min and subsequently cooled to room temperature. Finally, 200 mL of supernatant per tube was transferred to a 96-well microplate and the absorbance of the sample at 533 nm was measured.

### Measurement of Fe^2+^

Cells were seeded at a density of 1.5 × 10^5^ cells/well and cultured for 24 h at 37 °C and 5% CO_2_. Fe^2+^ was measured using Iron Assay kit (Abcam) according to the manufacturer’s instructions. The absorbance of the sample at 593 nm was measured by a reader from BioTek Instruments, Inc.

### Cell-death assays

Cells were seeded at a density of 3.5 × 10^3^ cells per well in 96-well plates. The following day, cells were treated with indicated concentrations of erastin as well as RSL3 or DMSO. After 48 h of incubation, cell viability was assayed by MTT at the absorbance of 570 nm. Cell death (%) was calculated using the following formula: (OD_control_ − OD_treated_) / OD_control_ × 100, where OD_control_ and OD_treated_ represent the absorbance of the control and treated groups, respectively.

### Protein stability and degradation analysis

Cycloheximide (CHX) has inhibitory effects on protein biosynthetic processes in eukaryotes. This impedes translation by interfering with translocation during protein synthesis. Approximately 30 h after transfection, AGS and SGC-7901 cells stably infected with RNF180 overexpression or control plasmids were incubated with cycloheximide (CHX, 50 µg/mL) to inhibit protein synthesis. Total protein was extracted for western blot analysis. MG132 is a proteasome inhibitor that inhibits protein degradation in a protease dependent manner. Therefore, 5 μM MG132 (hy12359; MedChemExpress) was added to AGS and SGC-7901 cells stably infected with RNF180 overexpression, and an equal amount of dimethyl sulfoxide (DMSO) was added to the control group. After 12 h of treatment, the total protein was extracted for western blot analysis.

### Statistical analysis

Statistical analyses were performed using IBM SPSS Statistics (version 25.0; Armonk, NY, USA) and the GraphPad Prism software (version 8.0). The P-value was calculated using the t-test, one-way analysis of variance, and two-way analysis of variance. OS was determined using the Kaplan–Meier method and log-rank test. The Cox proportional hazards model was used for multivariate analyses of OS. Statistical significance was set at P < 0.05.

## Supplementary Information

**Additional file 1: Fig S1.** The expression and the inhibition to proliferation of BCL6 on GC cells lines. A The protein expression levels of BCL6 in GC cells and immortalized normal gastric epithelial cell (GES-1) were examined by western blotting assay. B Expression levels of BCL6 in AGS and SGC-7901 cells infected with two BCL6 knockdown virus and empty virus were analyzed by western blotting assay (**p<0.01,***p<0.001). C BCL6 inhibits the colony formation of AGS and SGC-7901 cells (**p<0.01, ***p<0.001). **Fig S2.** Knockdown of BCL6 promotes malignant phenotypes in AGS and SGC-7901 cells. A, B, C Depletion of BCL6 promoted the viability and proliferation of AGS and SGC-7901 cells (Scale bar, 100μm) (***p<0.001). D Depletion of BCL6 promoted the migration of AGS and SGC-7901 cells (Scale bar, 200μm) (***p<0.001). E Depletion of BCL6 promoted the migration and invasion of AGS and SGC-7901 cells (Scale bar, 100μm) (**p<0.01, ***p<0.001). F Depletion of BCL6 promoted the EMT process of AGS and SGC-7901 cells. **Fig S3.** BCL6 inhibits the growth of GC cell. Tumor growth curves and tumor weight showed the inhibitory effect of BCL6 on tumor in vivo (N=8) (***p<0.001). **Fig S4.** BCL6 inhibits Wnt/β-catenin pathway by supressing FZD7. A β-catenin protein levels of GC cells after BCL6 knockdown combined with Wnt inhibitor IWP2 treatment (5 µM, 12h). B Representative immunohistochemistry of FZD7 from GC tissue microarrays (Original magnification, ×100 and ×400). C Representative immunohistochemistry of FZD7 and β-catenin in tumor xenografts derived from SGC-7901-NC, SGC-7901-BCL6 cell lines (Original magnification, ×400). D, E ChIP analysis of BCL6 binding to the FZD7 promoter in the 293T cells. The matched IgG was used as a negative control and vicinity region (VR) (-901bp―0 bp) also as a control (***p<0.001). F Western blot for nuclear and cytoplasmic β-catenin in AGS and SGC-7901 cells transfected with control vector (Ctrl1+Ctrl2, Ctrl1+FZD7, BCL6+Ctrl2) or co-transfected BCL6 plasmid and FZD7 plasmid (BCL6+FZD7). **Fig S5.** TP63 mediates the regulation of GPX4 by the BCL6/FZD7 pathway. A FZD7, TP63 and GPX4 mRNA levels in AGS and SGC-7901 cells transfected with control vector (Ctrl+shCtrl, Ctrl+shTP63, FZD7+shCtrl) or co-transfected FZD7 plasmid and shRNA targeting TP63 (FZD7+shTP63) (*p<0.05, **p<0.01, ***p<0.001). B BCL6, FZD7, TP63 and GPX4 mRNA levels in AGS and SGC-7901 cells transfected with control vector (Ctrl1+Ctrl2, Ctrl1+TP63, BCL6+Ctrl2) or co-transfected BCL6 plasmid and TP63 plasmid (BCL6+TP63) (*p<0.05, **p<0.01, ***p<0.001). **Fig S6.** IHC staining analysis of RNF180, RhoC and BCL6 in GC tissue microarrays. A Representative immunohistochemistry of RNF180 from GC tissue microarrays (Original magnification, ×100 and ×400). B Representative immunohistochemistry of RhoC from GC tissue microarrays (Original magnification, ×100 and ×400). C Correlations between the the expression of BCL6 and RhoC according to the IHC of TMAs. BCL6 negtively correlated with RhoC in human GC tissues (N=131). **Fig S7.** RhoC represses BCL6 expression in GC by reducing H3K27ac enrichment on its promoter through HDAC1. A Effects of RhoC on HDAC1, H3K9ac and H3K27ac in GC cells. B, C The HDAC1 inhibitor Pyroxamide (10μM, 24h) restores RhoC repression of H3K27ac and BCL6 (***p<0.001). D knockdown of RhoC significantly reduced the enrichment of HDAC1 on the promoter region of BCL6 (**p<0.01, ***p<0.001).E RhoC reduced H3K27ac enrichment on the BCL6 promoter region (**p<0.01, ***p<0.001). **Fig S8.** RNF180 promotes the expression of BCL6 in nucleus through RhoC and strengthened its inhibitory effect on downstream genes. A The upstream or downstream proteins expression of BCL6 in AGS and SGC-7901 cells transfected with control vector (Ctrl1+Ctrl2, Ctrl1+RhoC, RNF180+Ctrl2) or co-transfected RNF180 plasmid and RhoC plasmid (RNF180+RhoC). B Western blot for nuclear and cytoplasmic BCL6 in AGS and SGC-7901 cells transfected with control vector (Ctrl1+Ctrl2, Ctrl1+RhoC, RNF180+Ctrl2) or co-transfected RNF180 plasmid and RhoC plasmid RNF180+RhoC). **Fig S9.** Expression of BCL6 and its downstream genes is regulated by RNF180 and RhoC. A RNF180, RhoC, BCL6, FZD7, TP63, GPX4 mRNA expression in AGS and SGC-7901 cells transduced with control vector (Ctrl+Lenti-siCtrl, Ctrl+Lenti-siBCL6, RNF180+Lenti-siCtrl) or co-transduced RNF180 plasmid and Lenti-si BCL6 virus (RNF180+Lenti-siBCL6) (**p<0.01, ***p<0.001). B RhoC, BCL6, FZD7, TP63, GPX4 mRNA expression in AGS and SGC-7901 cells transduced with control vector (shCtrl+Lenti-siCtrl, shCtrl+Lenti-siBCL6, shRhoC+Lenti-siCtrl) or co-transduced shRhoC plasmid and Lenti-si BCL6 virus (shRhoC+ Lenti-siBCL6) (**p<0.01, ***p<0.001). **Fig S10.** Expression of BCL6 and its downstream proteins is regulated by RNF180 and RhoC. A RNF180, RhoC, HDAC1, H3K27ac, BCL6, FZD7, TP63, GPX4 protein expression in AGS and SGC-7901 cells transduced with control vector (Ctrl+Lenti-siCtrl, Ctrl+Lenti-siBCL6, RNF180+Lenti-siCtrl) or co-transduced RNF180 plasmid and Lenti-si BCL6 virus (RNF180+Lenti-siBCL6). B RhoC, HDAC1, H3K27ac, BCL6, FZD7, TP63, GPX4 protein expression in AGS and SGC-7901 cells transduced with control vector (shCtrl+Lenti-siCtrl, shCtrl+Lenti-siBCL6, shRhoC+Lenti-siCtrl) or co-transduced shRhoC plasmid and Lenti-si BCL6 virus (shRhoC+ Lenti-siBCL6). **Fig S11.** Malignancy reppression and ferroptosis facilitation of BCL6 in GC cells are up-regulated by RNF180. A, B BCL6 depletion reversed the inhibition of cell proliferation, migration and invasion induced by RNF180 overexpression in AGS and SGC-7901 cells(Scale bar, 100μm) (**p<0.01,***p<0.001). C, D BCL6 depletion reversed cell death induced by erastin or RSL3 in RNF180-overexpressing AGS and SGC-7901 cells (***p<0.001). **Table S1.** Correlation of BCL6 expression to clinicopathological features in gastric cancer patients. **Table S2.** The Primer Sequences Used in this Study.

**Additional file 2.** Other materials and methods.

## Data Availability

The Additional file 2 datasets used or analyzed during the current study are available from the corresponding author on reasonable request.

## Abbreviations

BCL6: B-cell lymphoma 6
TMD: Tumor maximum diameter
pN: Pathology node
FZD7: Frizzled class receptor 7
EMT: Epithelial–mesenchymal transformation
TP63: Tumor protein P63
GPX4: Glutathione peroxidase 4
GSH: Glutathione, r-glutamyl cysteingl glycine
ROS: Reactive oxygen species
RNF180: Ring finger protein 180
RhoC: Ras homolog gene family member C
CHX: Cycloheximide
GC: Gastric cancer
IHC: Immunohistochemistry
ChIP: Chromatin immunoprecipitation
BS: Binding site
VR: Vicinity region
