# Supplementary material for: The malignancy suppression and ferroptosis facilitation of BCL6 in gastric cancer mediated by FZD7 repression are strengthened by RNF180/RhoC pathway
Source: Cell Biosci. 2023 Apr 14;13:73. doi: 10.1186/s13578-023-01020-8 (PMC10105459; doi:10.1186/s13578-023-01020-8)
Supplement: Supplementary file 1 — Additional file 1: Fig S1. The expression and the inhibition to proliferation of BCL6 on GC cells lines. A The protein expression levels of BCL6 in GC cells and immortalized normal gastric epithelial cell (GES-1) were examined by western blotting assay. B Expression levels of BCL6 in AGS and SGC-7901 cells infected with two BCL6 knockdown virus and empty virus were analyzed by western blotting assay (**p<0.01,***p<0.001). C BCL6 inhibits the colony formation of AGS and SGC-7901 cells (**p<0.01, ***p<0.001). Fig S2. Knockdown of BCL6 promotes malignant phenotypes in AGS and SGC-7901 cells. A, B, C Depletion of BCL6 promoted the viability and proliferation of AGS and SGC-7901 cells (Scale bar, 100μm) (***p<0.001). D Depletion of BCL6 promoted the migration of AGS and SGC-7901 cells (Scale bar, 200μm) (***p<0.001). E Depletion of BCL6 promoted the migration and invasion of AGS and SGC-7901 cells (Scale bar, 100μm) (**p<0.01, ***p<0.001). F Depletion of BCL6 promoted the EMT process of AGS and SGC-7901 cells. Fig S3. BCL6 inhibits the growth of GC cell. Tumor growth curves and tumor weight showed the inhibitory effect of BCL6 on tumor in vivo (N=8) (***p<0.001). Fig S4. BCL6 inhibits Wnt/β-catenin pathway by supressing FZD7. A β-catenin protein levels of GC cells after BCL6 knockdown combined with Wnt inhibitor IWP2 treatment (5 µM, 12h). B Representative immunohistochemistry of FZD7 from GC tissue microarrays (Original magnification, ×100 and ×400). C Representative immunohistochemistry of FZD7 and β-catenin in tumor xenografts derived from SGC-7901-NC, SGC-7901-BCL6 cell lines (Original magnification, ×400). D, E ChIP analysis of BCL6 binding to the FZD7 promoter in the 293T cells. The matched IgG was used as a negative control and vicinity region (VR) (-901bp―0 bp) also as a control (***p<0.001). F Western blot for nuclear and cytoplasmic β-catenin in AGS and SGC-7901 cells transfected with control vector (Ctrl1+Ctrl2, Ctrl1+FZD7, BCL6+Ctrl2) or co-transfected BCL6 p [file 13578_2023_1020_MOESM1_ESM.docx]

**Additional file 1: figure legends：**


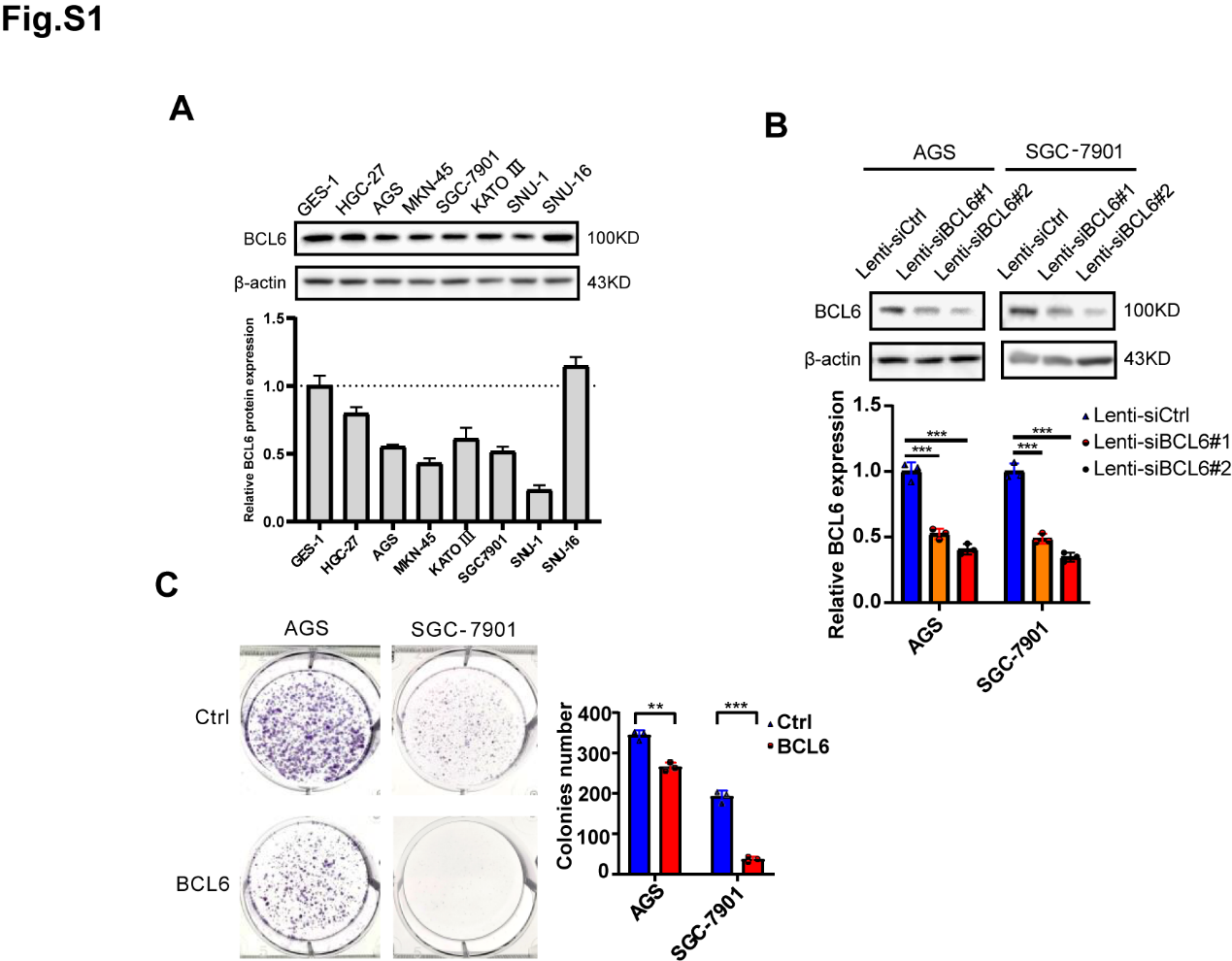


**Figure S1. The expression and the inhibition to proliferation of BCL6 on GC cells lines. A** The protein expression levels of BCL6 in GC cells and immortalized normal gastric epithelial cell (GES-1) were examined by western blotting assay. **B** Expression levels of BCL6 in AGS and SGC-7901 cells infected with two BCL6 knockdown virus and empty virus were analyzed by western blotting assay (**p<0.01,***p<0.001). **C** BCL6 inhibits the colony formation of AGS and SGC-7901 cells (**p<0.01,***p<0.001).


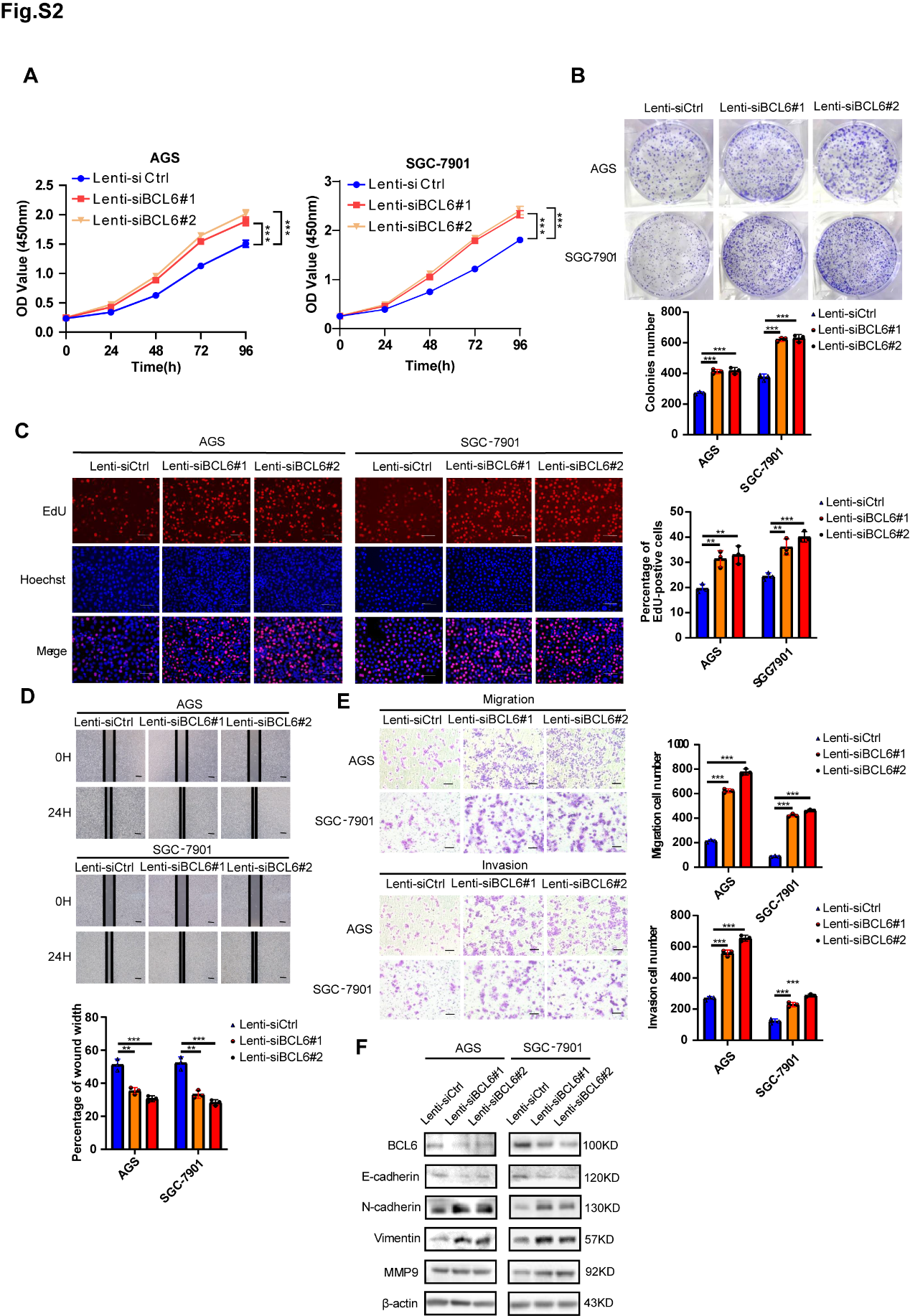


**Figure S2. Knockdown of BCL6 promotes malignant phenotypes in AGS and SGC-7901 cells. A, B, C** Depletion of BCL6 promoted the viability and proliferation of AGS and SGC-7901 cells (Scale bar, 100μm) (***p<0.001). **D** Depletion of BCL6 promoted the migration of AGS and SGC-7901 cells (Scale bar, 200μm) (***p<0.001). **E** Depletion of BCL6 promoted the migration and invasion of AGS and SGC-7901 cells (Scale bar, 100μm) (**p<0.01,***p<0.001). **F** Depletion of BCL6 promoted the EMT process of AGS and SGC-7901 cells.


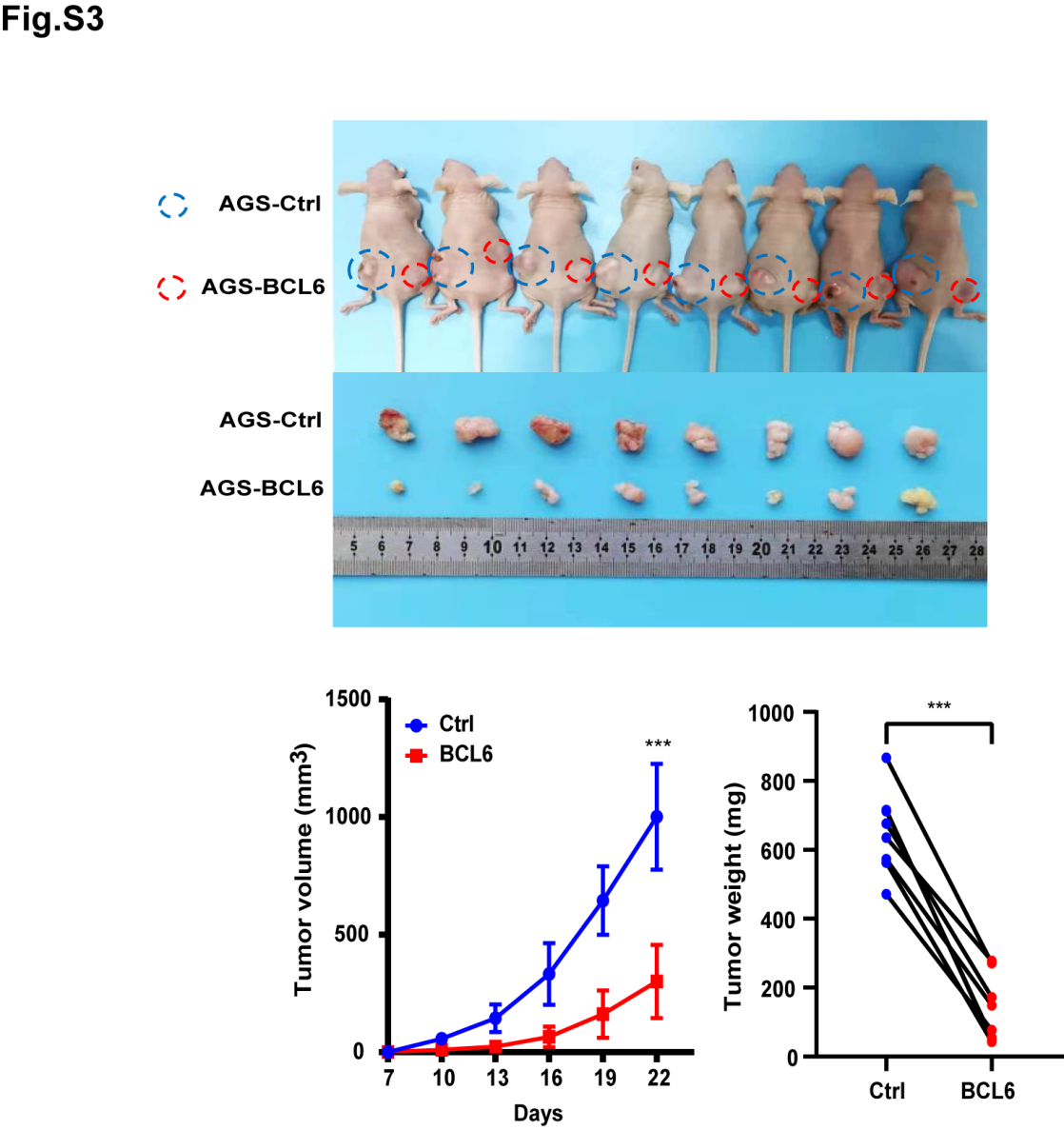


**Figure S3. BCL6 inhibits the growth of GC cell.** Tumor growth curves and tumor weight showed the inhibitory effect of BCL6 on tumor in vivo(N=8) (***p<0.001).


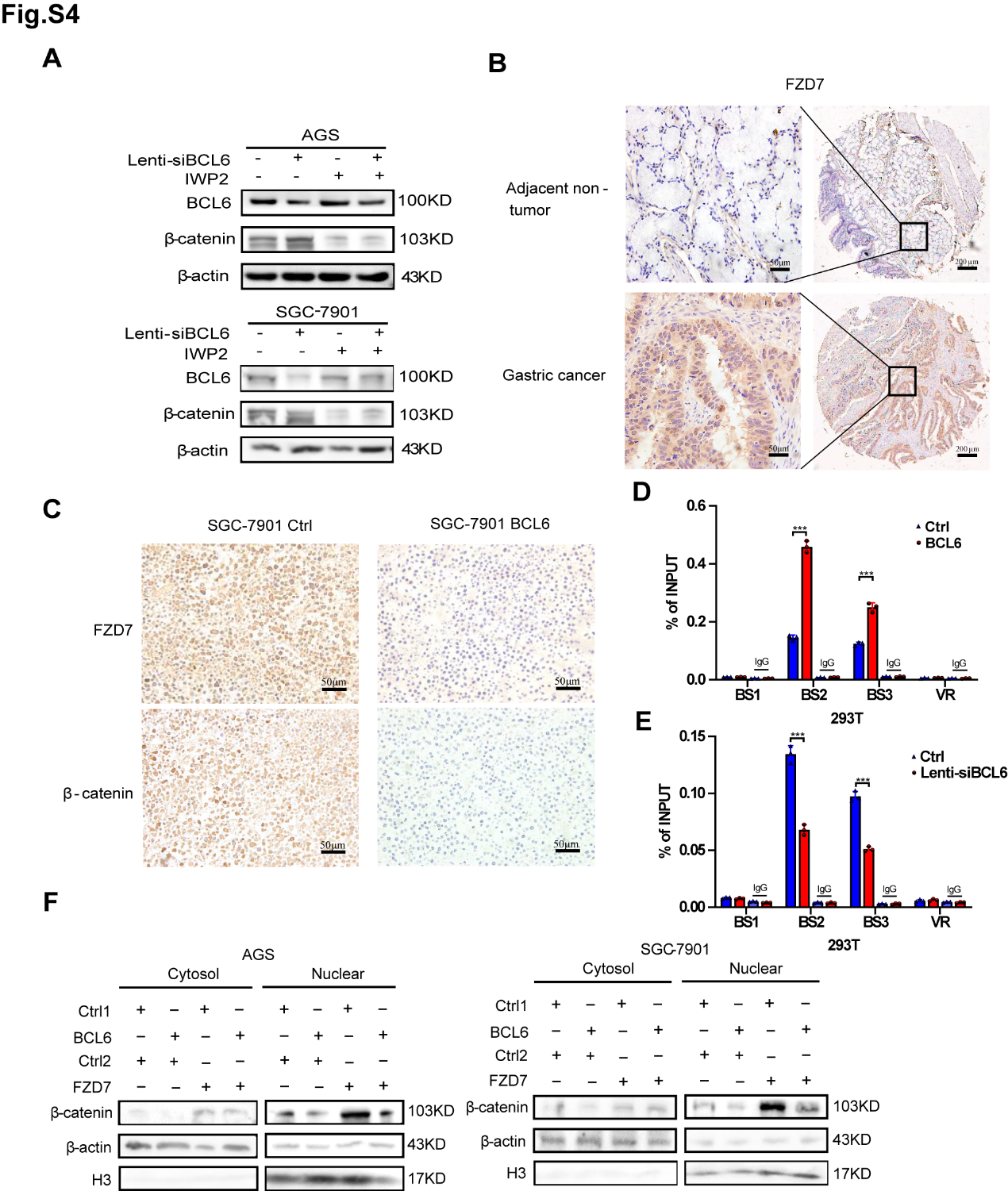


**Figure S4. BCL6 inhibits Wnt/β-catenin pathway by supressing FZD7**. **A** β-catenin protein levels of GC cells after BCL6 knockdown combined with Wnt inhibitor IWP2 treatment(5 µM,12h). **B** Representative immunohistochemistry of FZD7 from GC tissue microarrays (Original magnification, ×100 and ×400). **C** Representative immunohistochemistry of FZD7 and β-catenin in tumor xenografts derived from SGC-7901-NC, SGC-7901-BCL6 cell lines (Original magnification, ×400). **D, E** ChIP analysis of BCL6 binding to the FZD7 promoter in the 293T cells. The matched IgG was used as a negative control and vicinity region (VR) (-901bp―0bp) also as a control (***p<0.001). **F** Western blot for nuclear and cytoplasmic β-catenin in AGS and SGC-7901 cells transfected with control vector (Ctrl1+Ctrl2, Ctrl1+FZD7, BCL6+Ctrl2) or co-transfected BCL6 plasmid and FZD7 plasmid (BCL6+FZD7).


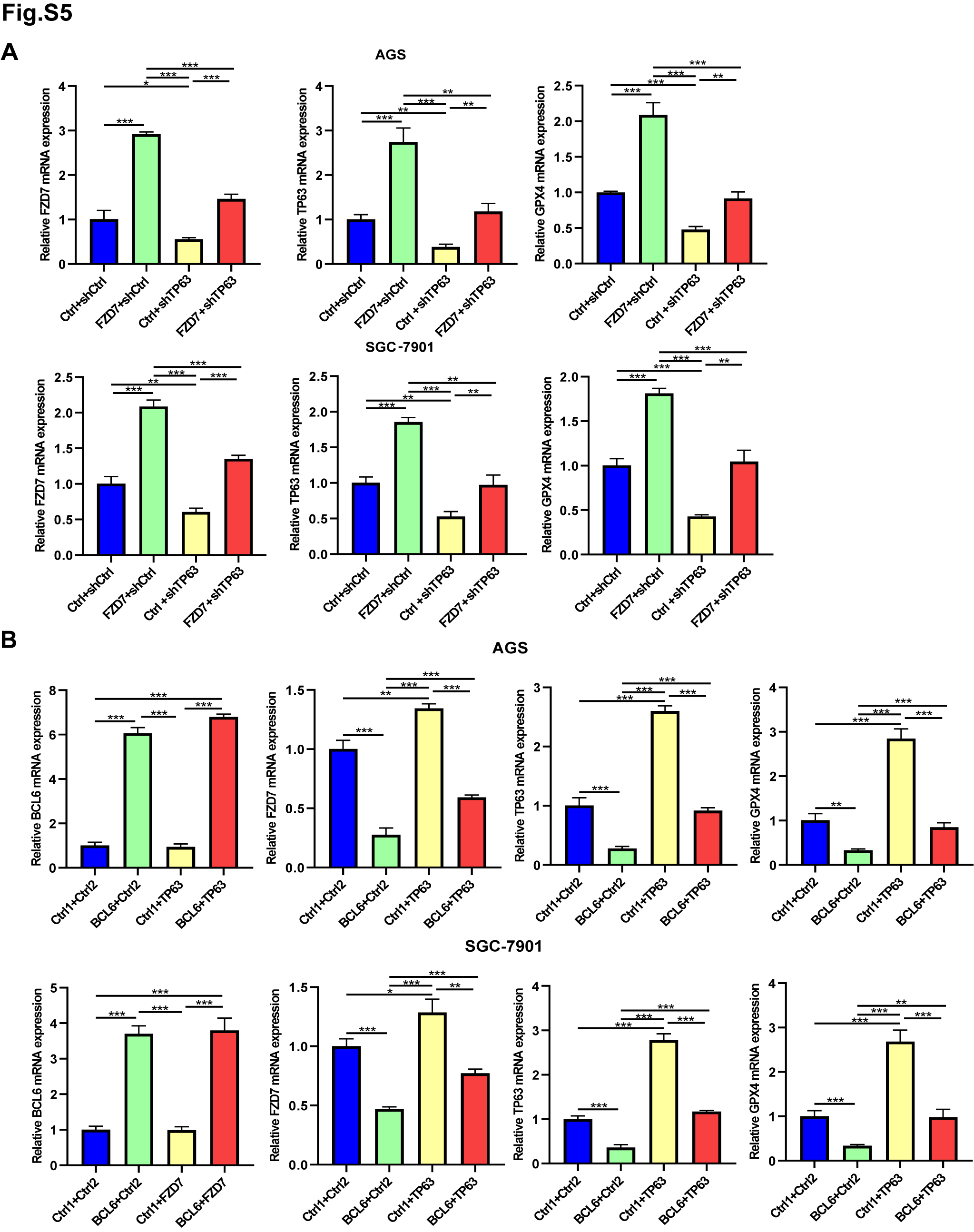


**Figure S5. TP63 mediates the regulation of GPX4 by the BCL6/FZD7 pathway.** **A** FZD7, TP63 and GPX4 mRNA levels in AGS and SGC-7901 cells transfected with control vector (Ctrl+shCtrl, Ctrl+shTP63, FZD7+shCtrl) or co-transfected FZD7 plasmid and shRNA targeting TP63 (FZD7+shTP63) (*p<0.05, **p<0.01, ***p<0.001). **B** BCL6, FZD7, TP63 and GPX4 mRNA levels in AGS and SGC-7901 cells transfected with control vector (Ctrl1+Ctrl2, Ctrl1+TP63, BCL6+Ctrl2) or co-transfected BCL6 plasmid and TP63 plasmid (BCL6+TP63) (*p<0.05, **p<0.01, ***p<0.001).


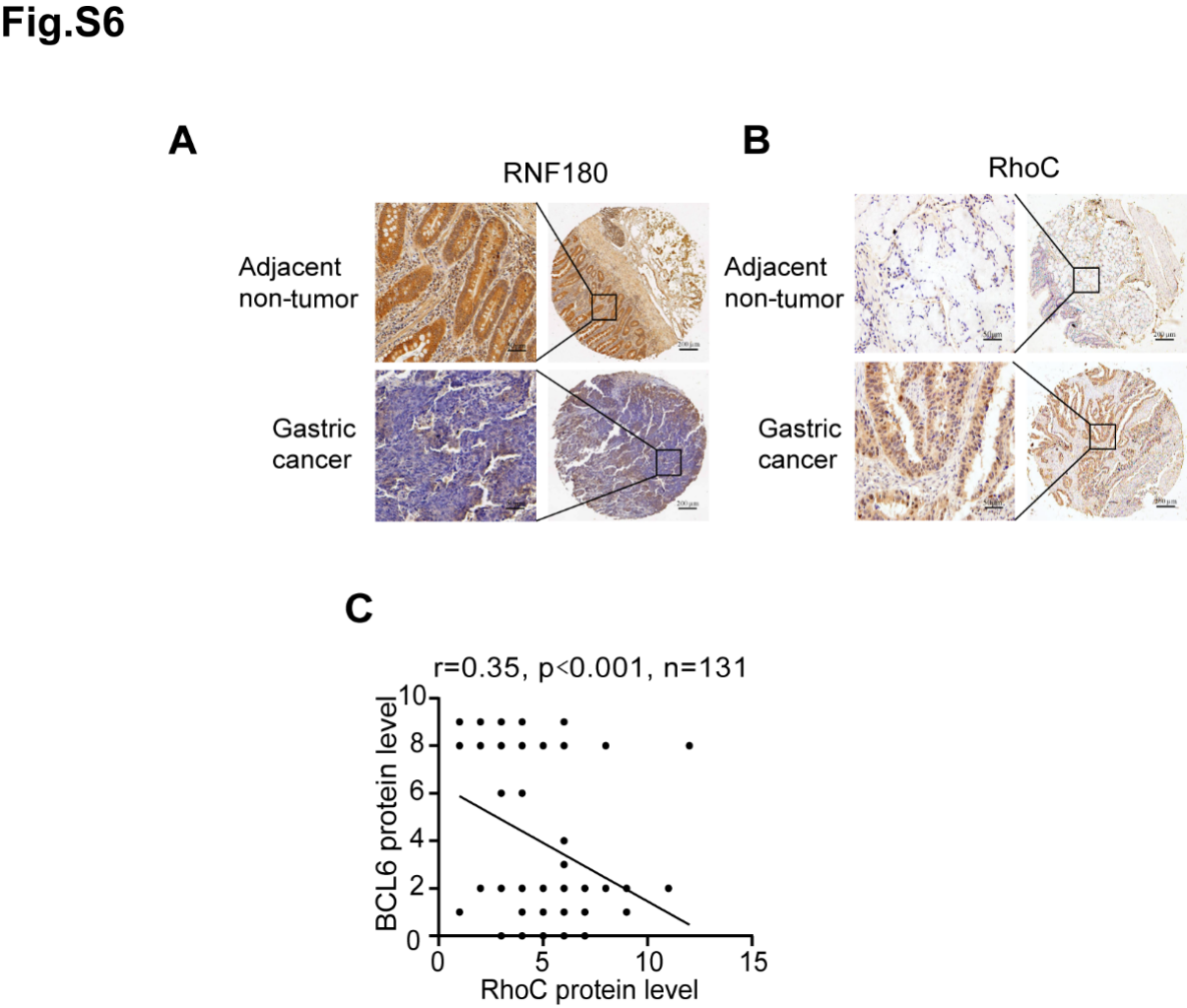


**Figure S6. IHC staining analysis of RNF180, RhoC and BCL6 in GC tissue microarrays**

**A** Representative immunohistochemistry of RNF180 from GC tissue microarrays (Original magnification, ×100 and ×400). **B** Representative immunohistochemistry of RhoC from GC tissue microarrays (Original magnification, ×100 and ×400). **C** Correlations between the the expression of BCL6 and RhoC according to the IHC of TMAs. BCL6 negtively correlated with RhoC in human GC tissues (N=131).


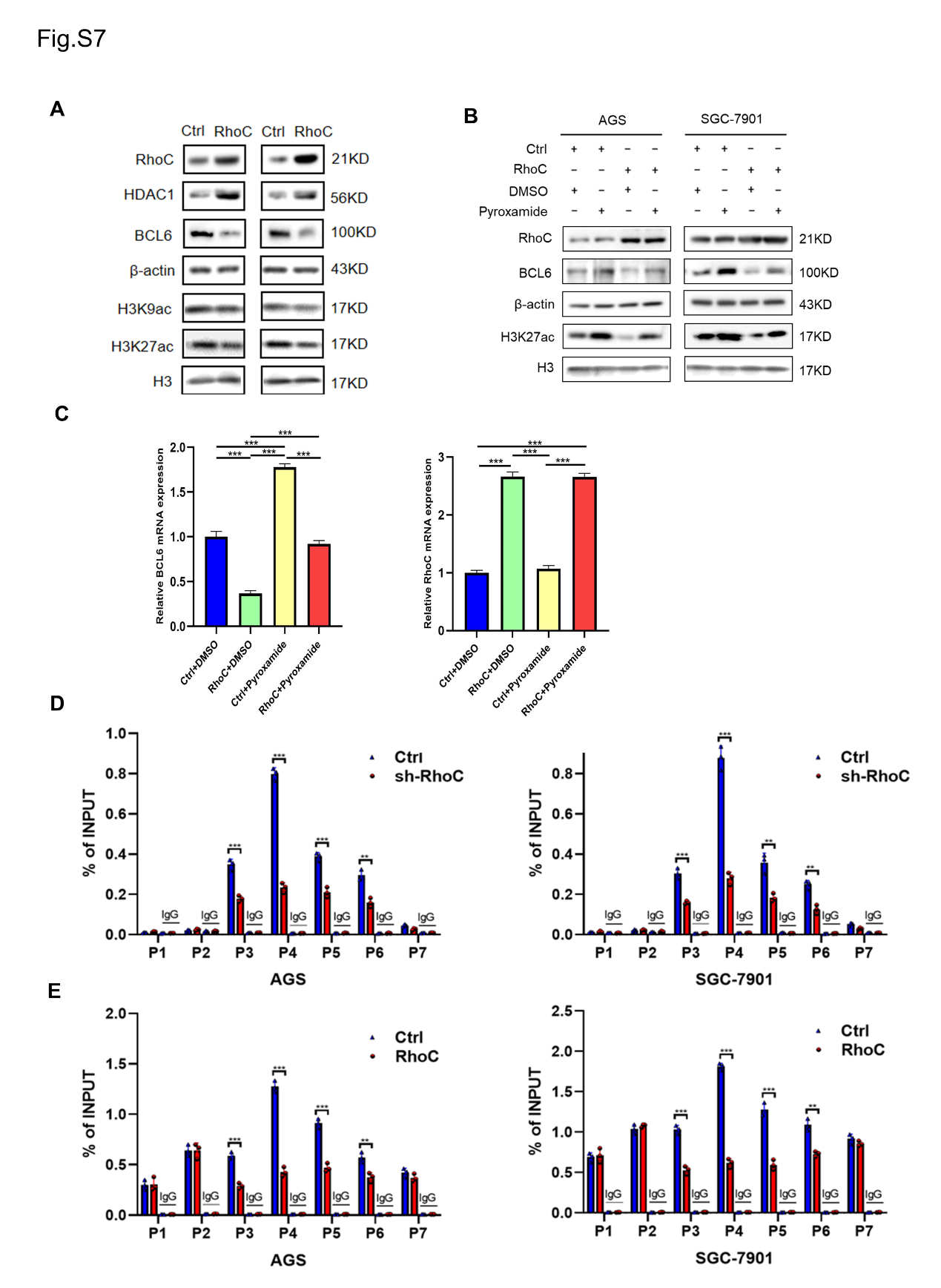


**Figure S7. RhoC represses BCL6 expression in GC by reducing H3K27ac enrichment on its promoter through HDAC1. A** Effects of RhoC on HDAC1, H3K9ac and H3K27ac in GC cells. **B, C** The HDAC1 inhibitor Pyroxamide (10μM,24h) restores RhoC repression of H3K27ac and BCL6 (***p<0.001). **D** knockdown of RhoC significantly reduced the enrichment of HDAC1 on the promoter region of BCL6 (**p<0.01,***p<0.001).**E** RhoC reduced H3K27ac enrichment on the BCL6 promoter region (**p<0.01,***p<0.001).


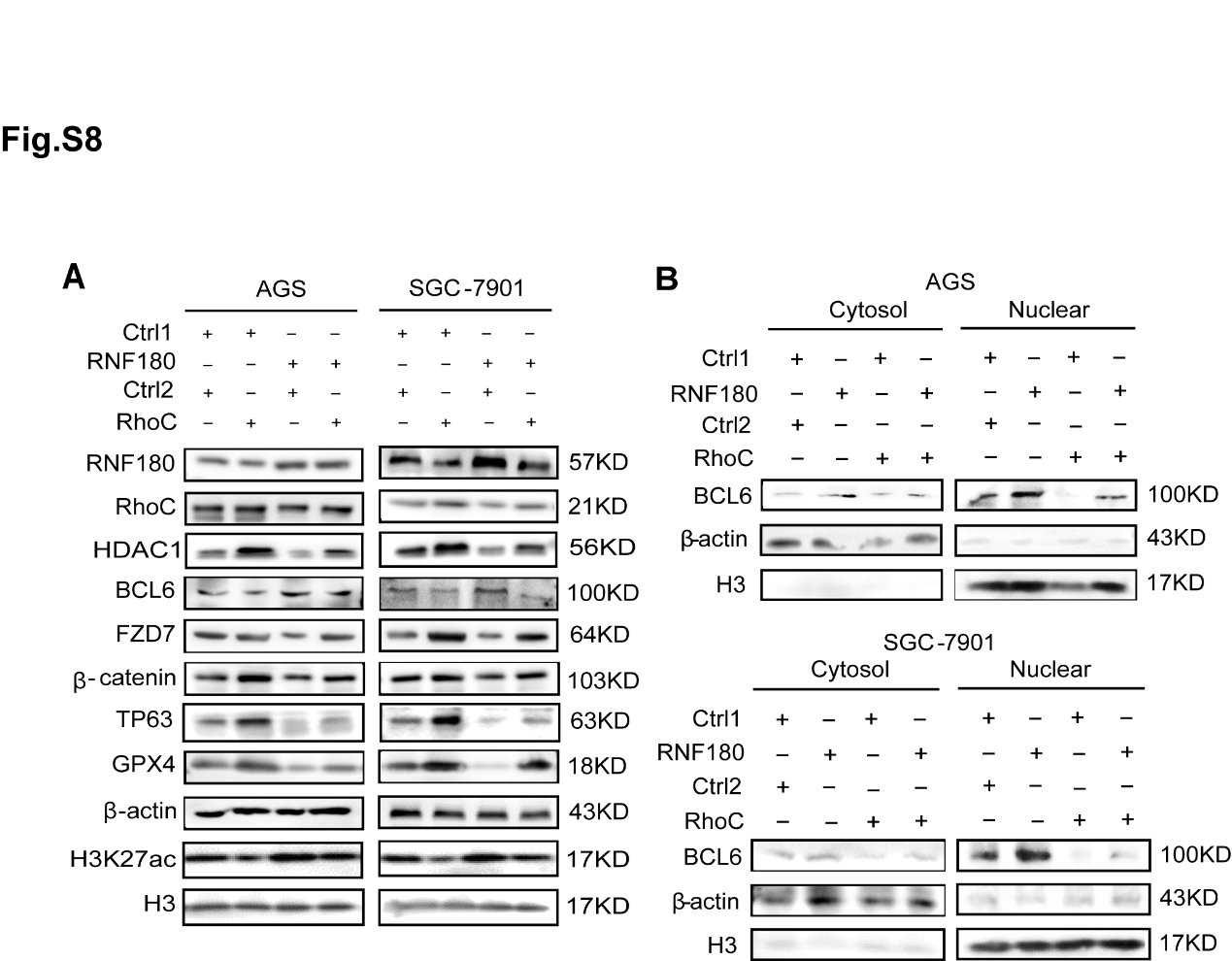


**Figure S8. RNF180 promotes the expression of BCL6 in nucleus through RhoC and strengthened its inhibitory effect on downstream genes.**

**A** The upstream or downstream proteins expression of BCL6 in AGS and SGC-7901 cells transfected with control vector (Ctrl1+Ctrl2, Ctrl1+RhoC, RNF180+Ctrl2) or co-transfected RNF180 plasmid and RhoC plasmid (RNF180+RhoC). **B** Western blot for nuclear and cytoplasmic BCL6 in AGS and SGC-7901 cells transfected with control vector (Ctrl1+Ctrl2, Ctrl1+RhoC, RNF180+Ctrl2) or co-transfected RNF180 plasmid and RhoC plasmid RNF180+RhoC).

**
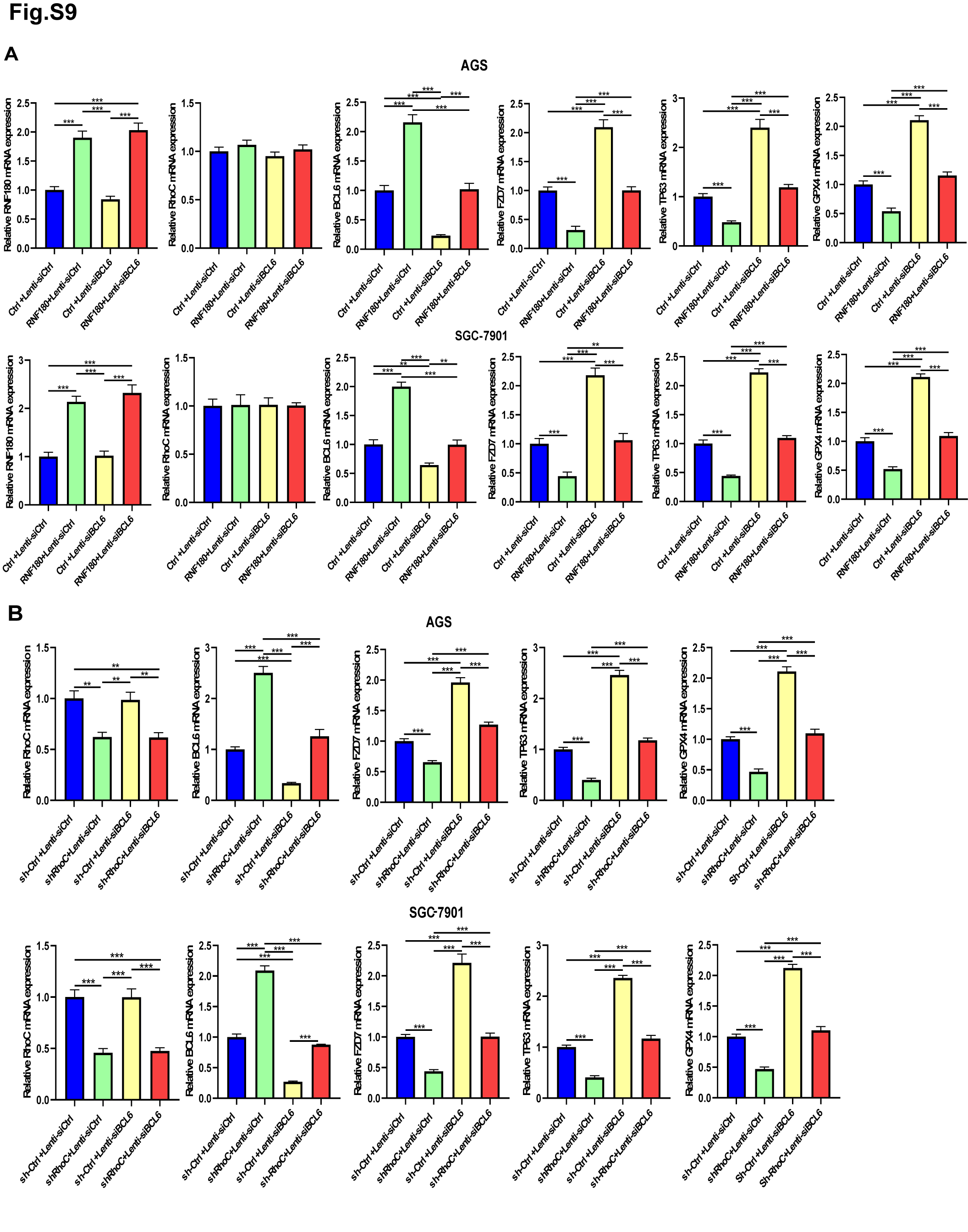
Figure S9. Expression of BCL6 and its downstream genes is regulated by RNF180 and RhoC. A** RNF180, RhoC, BCL6, FZD7, TP63, GPX4 mRNA expression in AGS and SGC-7901 cells transduced with control vector (Ctrl+Lenti-siCtrl, Ctrl+Lenti-siBCL6, RNF180+Lenti-siCtrl) or co-transduced RNF180 plasmid and Lenti-si BCL6 virus (RNF180+Lenti-siBCL6) (**p<0.01, ***p<0.001). **B** RhoC, BCL6, FZD7, TP63, GPX4 mRNA expression in AGS and SGC-7901 cells transduced with control vector (shCtrl+Lenti-siCtrl, shCtrl+Lenti-siBCL6, shRhoC+Lenti-siCtrl) or co-transduced shRhoC plasmid and Lenti-si BCL6 virus (shRhoC+ Lenti-siBCL6) (**p<0.01, ***p<0.001).


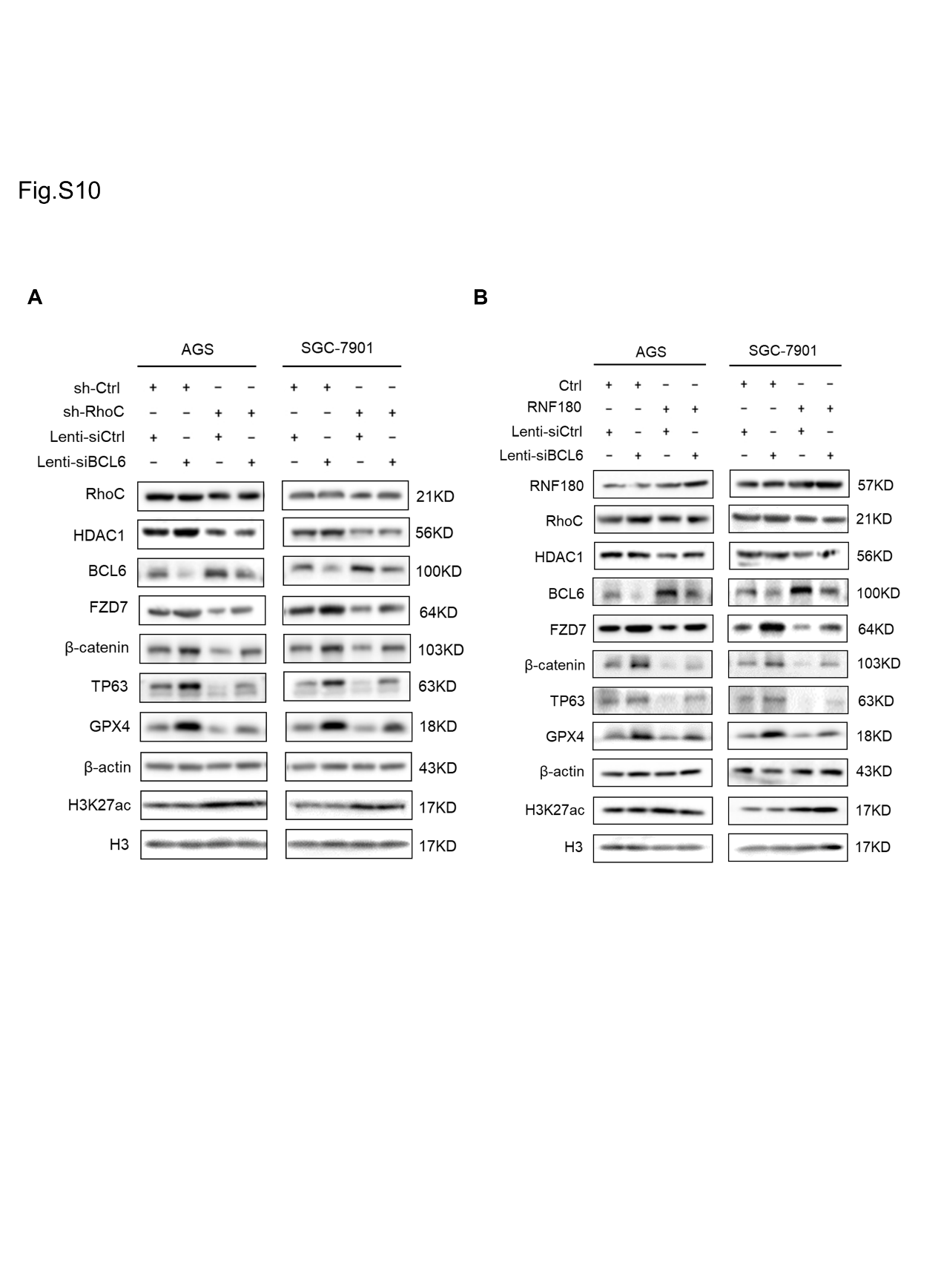


**Figure S10. Expression of BCL6 and its downstream proteins is regulated by RNF180 and RhoC. A** RNF180, RhoC, HDAC1, H3K27ac, BCL6, FZD7, TP63, GPX4 protein expression in AGS and SGC-7901 cells transduced with control vector (Ctrl+Lenti-siCtrl, Ctrl+Lenti-siBCL6, RNF180+Lenti-siCtrl) or co-transduced RNF180 plasmid and Lenti-si BCL6 virus (RNF180+Lenti-siBCL6). **B** RhoC, HDAC1, H3K27ac, BCL6, FZD7, TP63, GPX4 protein expression in AGS and SGC-7901 cells transduced with control vector (shCtrl+Lenti-siCtrl, shCtrl+Lenti-siBCL6, shRhoC+Lenti-siCtrl) or co-transduced shRhoC plasmid and Lenti-si BCL6 virus (shRhoC+ Lenti-siBCL6).


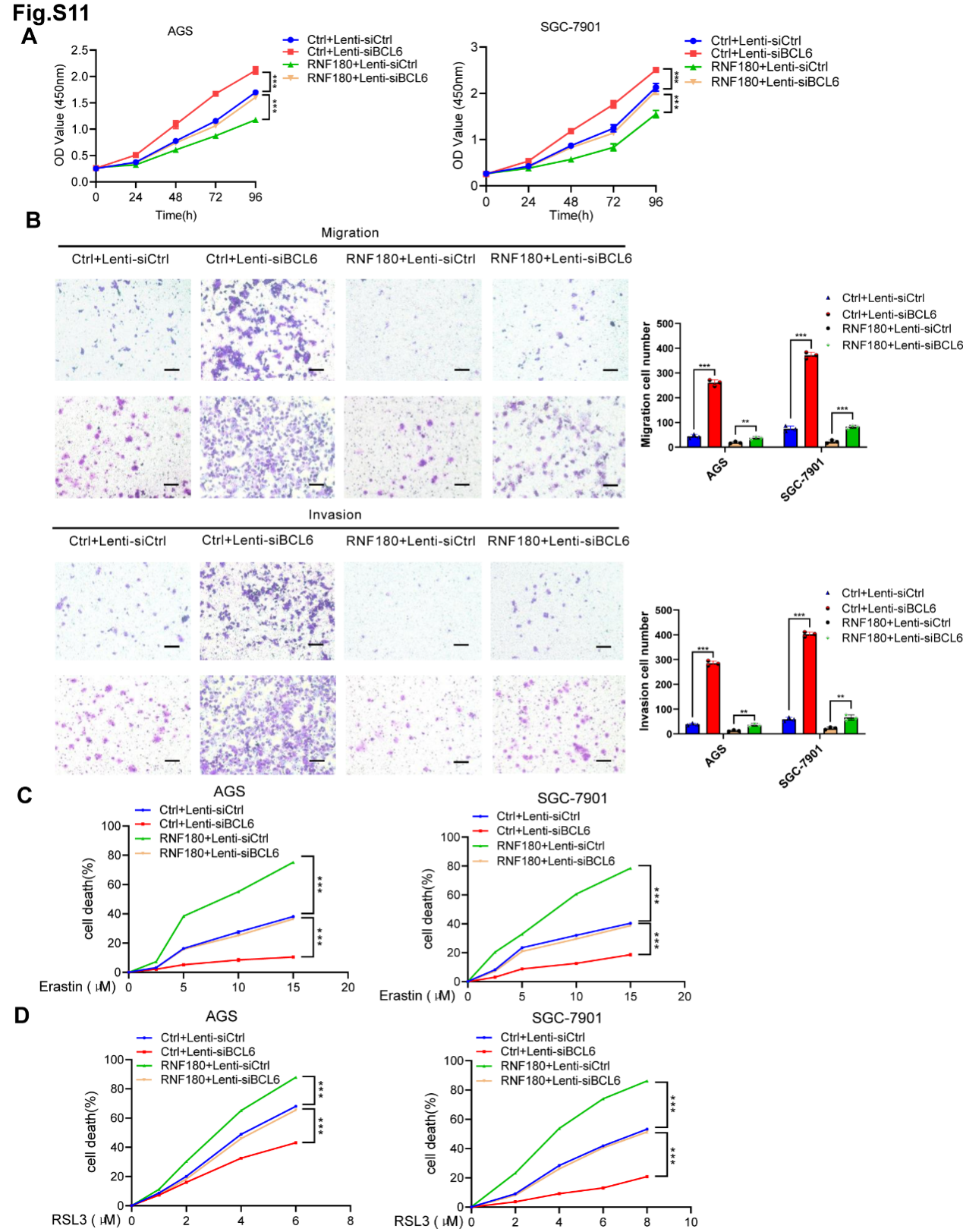


**Figure S11. Malignancy reppression and ferroptosis facilitation of BCL6 in GC cells are up-regulated by RNF180. A, B** BCL6 depletion reversed the inhibition of cell proliferation, migration and invasion induced by RNF180 overexpression in AGS and SGC-7901 cells(Scale bar, 100μm) (**p<0.01,***p<0.001). **C, D** BCL6 depletion reversed cell death induced by erastin or RSL3 in RNF180-overexpressing AGS and SGC-7901 cells (***p<0.001).

**Additional file 1: Table legends:**

**Table S1. Correlation of BCL6 expression to clinicopathological features in gastric cancer patients**

| Characteristics Total | | BCL6 expression N (%） | | χ2 | P-value |
| --- | --- | --- | --- | --- | --- |
|  |  | Low | High |  |  |
| Gender |  |  |  | 1.817 | 0.232 |
| Male | 98 | 61(62.2) | 37(37.8) |  |  |
| Female | 39 | 29(74.4) | 10(25.6) |  |  |
| Age(year) |  |  |  | 0.310 | 0.710 |
| ＜65 | 86 | 55(64.0) | 31(36.0) |  |  |
| ≥65 | 51 | 35(68.6) | 16(31.4) |  |  |
| pT stage |  |  |  | 3.352 | 0.193a |
| pT2 | 10 | 5(50.0) | 5(50.0) |  |  |
| pT3 | 9 | 4(44.4) | 5(55.6) |  |  |
| pT4 | 118 | 81(68.6) | 37(31.4) |  |  |
| pN stage |  |  |  | 10.690 | 0 .013* |
| pN0 | 31 | 16(51.6) | 15(48.4) |  |  |
| pN1 | 14 | 8(57.1) | 6(42.9) |  |  |
| pN2 | 34 | 19(55.9) | 15(44.1) |  |  |
| pN3 | 58 | 47(81.0) | 11(19.0) |  |  |
| Tumor location |  |  |  | 2.301 | 0.536 |
| Upper third | 20 | 11(55.0) | 9(45.0) |  |  |
| Middle third | 18 | 13(72.2) | 5(27.8) |  |  |
| Lower third | 72 | 46(63.9) | 26(36.1) |  |  |
| More than 2/3 stoma | 27 | 20(74.1) | 7(25.9) |  |  |
| Tumor size(cm) |  |  |  | 4.356 | 0 .041* |
| <5 | 48 | 26(54.2) | 22(45.8) |  |  |
| ≥5 | 89 | 64(71.9) | 25(28.1) |  |  |
| Lauren type |  |  |  | 1.896 | 0.327a |
| Intestinal | 32 | 20(62.5) | 12(37.5) |  |  |
| Diffuse | 102 | 69(67.6) | 33(32.4) |  |  |
| Mixed | 3 | 1(33.3) | 2(66.7) |  |  |
| Bormann type |  |  |  | 2.431 | 0.600a |
| Ⅰ | 2 | 2(100.0) | 0(0.0) |  |  |
| Ⅱ | 40 | 27(67.5) | 13(32.5) |  |  |
| Ⅲ | 85 | 53(62.4) | 32(37.6) |  |  |
| Ⅳ | 10 | 8(80.0) | 2(20.0) |  |  |

a fisher exact test;

* P<0 . 05

**Table S2. The Primer Sequences Used in this Study**

| Gene | type | Sequence (5’ to 3’) |
| --- | --- | --- |
| BCL6 | Forward primer | GTCGTGAGGTGGTGGAGAACAAC |
| BCL6 | Reverse primer | AGAGGCTGGCGGTGTGGAC |
| FZD7 | Forward primer | GTCAAGACCATCACTATCCTGG |
| FZD7 | Reverse primer | ATGAAGAGGTAGACGAACAGAG |
| TP63 | Forward primer | GCAACGCCCTCACTCCTACAAC |
| TP63 | Reverse primer | AGTCCATTCATGTCTCCAGCCATTG |
| GPX4 | Forward primer | CCCGATACGCTGAGTGTGGTTTG |
| GPX4 | Reverse primer | TCTTCGTTACTCCCTGGCTCCTG |
| RNF180 | Forward primer | TGTTACCCTTGCCATCACATCTTCT |
| RNF180 | Reverse primer | CCATCCACGGCTATCATCCTCAAA |
| RhoC | Forward primer | AGGTCTACGTCCCTACTGTCTTTGA |
| RhoC | Reverse primer | GTCTTGCCTCAGGTCCTTCTTATTC |
| β-actin | Forward primer | CATGTACGTTGCTATCCAGGC |
| β-actin | Reverse primer | CTCCTTAATGTCACGCACGAT |
| BCL6/FZD7 binding site 1 | Forward primer | GGAGGGCGAAACTGATGCTTGG |
| BCL6/FZD7 binding site 1 | Reverse primer | GTTAATAGGTCGAAAGGGCGGAAGG |
| BCL6/FZD7 binding site 2 | Forward primer | CCTGCTGTAGAAGAAGCGCA |
| BCL6/FZD7 binding site 2 | Reverse primer | TGGAGCTCTGAGCGATTTCTG |
| BCL6/FZD7 binding site 3 | Forward primer | CGAGGATAACCCCGCTTTGT |
| BCL6/FZD7 binding site 3 | Reverse primer | AGGAACACTCTGTACGACGC |
| BCL6/FZD7 Vicinity region | Forward primer | AGTGCACCAGGAAGAGGAACAAATC |
| BCL6/FZD7 Vicinity region | Reverse primer | TGGGAGGGAAAAGTTCGGAAAAACT |
| FZD7 promoter–2000/+180 | Forward primer | GGGGTACCCAACCGAAGATCAAC |
| FZD7 promoter–2000/+180 | Reverse primer | CCCAAGCTTACAGTCAAGGCATTC |
| FZD7 promoter –1672/+180 | Forward primer | GGGGTACCGGAAAGAACGATTCC |
| FZD7 promoter –1672/+180 | Reverse primer | CCCAAGCTTACAGTCAAGGCATTC |
| FZD7 promoter –1296/+180 | Forward primer | GGGGTACCCAACATGTCACTTTC |
| FZD7 promoter –1296/+180 | Reverse primer | CCCAAGCTTACAGTCAAGGCATTC |
| FZD7 promoter –950/+180 | Forward primer | GGGGTACCAGGCACACGGGGCCA |
| FZD7 promoter –950/+180 | Reverse primer | CCCAAGCTTACAGTCAAGGCATTC |
| FZD7 promoter BS1 mutation | Forward primer | GGCGAAACTGACACACACACACACACAGGCGA |
| FZD7 promoter BS1 mutation | Reverse primer | GGGAGTCGCCTGTGTGTGTGTGTGTGTCAGTT |
| FZD7 promoter BS2 mutation | Forward primer | GAATGAGTTTTTTTCACACACACACACAGCACG |
| FZD7 promoter BS2 mutation | Reverse primer | TGCCTCCCGTGCTGTGTGTGTGTGTGAAAA |
| FZD7 promoter BS3 mutation | Forward primer | ACTCTTTAATTAGCCACACACACACACACACATTAA |
| FZD7 promoter BS3 mutation | Reverse primer | TAGGAATGTTTTAATGTGTGTGTGTGTGTGTGGCTA |
| BCL6-P1 -2000/-1687 | Forward primer | TGGAACTTCTTACGGGACTGTC |
| BCL6-P1 -2000/-1687 | Reverse primer | AATGGAGGAGTGATCAGAGCTG |
| BCL6-P2 -1686/-1373 | Forward primer | GATTCTGCACTGCAATTGCC |
| BCL6-P2 -1686/-1373 | Reverse primer | CACGCAGCACAGTCTTCTTAG |
| BCL6-P3 -1372/-1059 | Forward primer | CCTTTAGAATTCCCTCCGGCTGATC |
| BCL6-P3 -1372/-1059 | Reverse primer | TTTGTTTTCGAGGCTCGTTTCAAGG |
| BCL6-P4 -1058/-745 | Forward primer | GGGGCTGGGATTGTTACATATGGC |
| BCL6-P4 -1058/-745 | Reverse primer | AATCACAGGATCGGCATCGGTTG |
| BCL6-P5 -744/-431 | Forward primer | CCCAAGCCACCACACCAGAAAG |
| BCL6-P5 -744/-431 | Reverse primer | AAGCAGTTTTCGGTGGGTTCATTTG |
| BCL6-P6 -430/-117 | Forward primer | GAGCAGGCCATACCATCGTCTTG |
| BCL6-P6 -430/-117 | Reverse primer | TCATAAGCAGAGCGTCTCGGATTTG |
| BCL6-P7 -116/+200 | Forward primer | TGTTGATTCTTCTGGCTTGGTCTGG |
| BCL6-P7 -116/+200 | Reverse primer | CACTAGGTTAGGAGCTGAGGGAGTC |
