# Supplementary material for: The malignancy suppression and ferroptosis facilitation of BCL6 in gastric cancer mediated by FZD7 repression are strengthened by RNF180/RhoC pathway
Source: Cell Biosci. 2023 Apr 14;13:73. doi: 10.1186/s13578-023-01020-8 (PMC10105459; doi:10.1186/s13578-023-01020-8)
Supplement: Supplementary file 2 — Additional file 2. Other materials and methods. [file 13578_2023_1020_MOESM2_ESM.docx]

**Other materials and methods:**

**Cell culture and reagents**

The AGS GC cell line was acquired from American Type Culture Collection (ATCC, Manassas, VA, USA). The SGC-7901 cell line was obtained from Beijing Cancer Institute, Peking University, China. AGS cells were cultured in an F12 nutrient mixture medium (GIBCO, Carlsbad, CA, USA) supplemented with 10% fetal bovine serum. In addition, SGC-7901 cells were cultured in RPMI 1640 medium supplemented with 10% fetal bovine serum (GIBCO, Carlsbad, CA, USA). All cell lines were cultured at 37℃ in an atmosphere of 5% CO_2_. The Wnt palmitoyltransferase inhibitor (IWP-2) was purchased from Sigma Aldrich (St. Louis, MO, USA), and was added to the cells at 5 µM concentration.

**Western blotting**

Western blotting was performed according to the standard methods. The primary antibodies used for western blotting were as follows: rabbit anti-RNF180 antibody (1:1000) (gtx119301, Genetex), mouse anti-RhoC antibody (1:50) (sc393090, Santa Cruz Biotechnology), rabbit anti-BCL6 antibody (1:200) ([14895](https://www.cellsignal.cn/products/primary-antibodies/bcl6-d4i2v-xp-rabbit-mab/14895?site-search-type=Products&N=4294956287&Ntt=bcl6&fromPage=plp)s, CST), rabbit anti-BCL6 antibody (1:1000) (ab172610, abcam), mouse anti-FZD7 antibody (1:200) (sc-293261, Santa Cruz Biotechnology), rabbit anti-E-cadherin antibody (1:2000) (3195, Cell Signaling Technology), rabbit anti-N-cadherin antibody (1:2000) (4061s, Cell Signaling Technology), rabbit anti-vimentin antibody (1:1000) (ab20346, Abcam), rabbit anti-MMP9 antibody (1:2000) (3852s, Cell Signaling Technology), rabbit anti-β-catenin antibody (1:5000) (ab16051, Abcam), mouse anti-β-catenin antibody (1:1000) (NBP1-51588, Novus), rabbit anti-TP63 antibody (1:1000) (ab124726, Abcam),rabbit anti-GPX4 antibody (1:1000) (ab125066, Abcam), and rabbit anti-β-actin antibody (1:1000) (gtx109639, Genetex), rabbit anti-HDAC1 (1:1000) (ab109411, Abcam), rabbit anti-H3K27ac antibody (1:1000) (8173s, Cell Signaling Technology), rabbit anti-H3K9ac antibody (1:1000) (9649s, Cell Signaling Technology), and rabbit anti-H3 antibody (1:1000) (4620s, Cell Signaling Technology).

**RNA extraction and PCR**

Total RNA was extracted using the TRIzol reagent (Invitrogen, CA, USA). Complementary DNA was synthesized using PrimeScript™ RT Master Mix (TaKaRa, Shiga, Japan) according to the manufacturer’s instructions. For semi-quantitative reverse transcription PCR, the *RhoC, BCL6*, and *β-actin* genes were amplified using a 2X Taq PCR master mix (Tiangen Biotech, Beijing, China) according to the manufacturer’s protocol. PCR for the *RhoC* gene was carried out under the following conditions: 3 min at 94 °C, 35 cycles of 30 s at 94 °C, 30 s at 53 °C, and 1 min at 72 °C, and a final 5 min at 72 °C. The PCR for *BCL6* and *β-actin* gene was carried out under the same conditions but at 61°C and 59°C annealing temperatures, respectively. Primers designed and used for RhoC were 5'- AGGTCTACGTCCCTACTGTCTTTGAG-3' and 3'-GTCTTGCCTCAGGTCCTTCTTATTCC-5'. The primers used for BCL6 were 5'- GTGTGACTGCCGCTTCTCTGAG-3' and 3'- GTGGATTCGCAGGTGGCTCTTC-5'. SYBR Green PCR Master Mix (Takara, Shiga, Japan) was used for qPCR. RT-qPCR was performed as previously described. The 2^−ΔΔCT^ method was used for data analysis, and β-actin was used as the internal control. The rest of primers used are listed in Supplementary Table S2.

**Plasmids, short hairpin RNA, and lentiviruses**

The expression level of RNF180 was up-regulated by the plasmid (PCMV6-AC-GFP-RNF180). RhoC was overexpressed by the plasmid (PCMV6-AC-GFP-RhoC). BCL6 was overexpressed by the plasmid (PLVX-BCL6). FZD7 was overexpressed by the plasmid (FV249/CMV-MCS-IRES-DsRED-SV40-NeoR-FZD7). FZD7 promoter luciferase reporters were constructed by inserting promoter fragments, PCR-amplified from genomic DNA, into the pGL3-Basic vector. All mutations were introduced in pGL3 luciferase plasmids, which were generated by site-directed mutagenesis using PCR. TP63 was overexpressed by the plasmid (PCMV6-AC-GFP-TP63). Plasmids were transfected using the Lipofectamine^TM^ 3000 transfection reagent (Invitrogen, New York, USA) and Opti-MEM (Invitrogen, New York, USA). RhoC and TP63 were knocked down with short hairpin RNA (shRNA) obtained from Shanghai GeneChem Co. (Shanghai, China) and liposome amine 3000 was used to transfect shRNA into GC cells. Simultaneously, empty control vectors were transfected into the cells. The suitable cell lines AGS , SGC-7901 GC cells were screened from various cancer cells for lentivirus infection. AGS, SGC-7901 and 293T cells were infected with BCL6-overexpression or two knocking down lentivirus and their control lentivirus vector obtained from KaiJi (NanJing, China). Cells were infected by lentivirus with 3 μg/mL polybrene for three days. Stably expressing BCL6 cell lines were selected using puromycin treatment. Transfection or infection efficiency was determined using qPCR or western blotting. The Lenti-siBCL6 with the highest knockdown efficiency was selected for subsequent cell function recovery assay.

**Cell viability assay**

Cell viability was evaluated using the Cell Counting Kit 8 (CCK8). AGS and SGC-7901 GC cells (1×10^3^) infected with the designated lentivirus, plasmid, and/or shRNA were seeded into each well of 96-well plates. CCK8 (10 μL) was added to each well once a day and incubated at 37 ℃ for 2 h. Then, the optical density at 450 nm wavelength was measured using a spectrophotometer. This assay was repeated three times.

**Colony formation assay**

The proliferative ability of GC cells was detected using a colony formation assay. First, 1×10^3^ AGS and SGC-7901 GC cells infected with designated lentivirus, plasmid, and/or shRNA were seeded into each well of 96-well plates and cultured at 37°C for 12–14 days. Next, the medium was changed regularly until the clones were visible to the naked eye. The cell colonies were then stained with crystal violet solution (0.005% crystal violet; 25% methanol) and further quantified. This assay was repeated three times.

**EdU assay**

For the EdU assay, cells (1.0×10^5^ cells / well) were plated into 24-well plates, and cultured at 37°C for 24 h. Cells were stained with the BeyoClick EdU-594 kit (Beyotime), nuclei with Hoechst, and images were obtained using an fluorescence microscope (Leica). The percentage of EdU-positive cells was quantified using ImageJ software.

**Wound healing assay**

AGS and SGC-7901 GC cells infected with the designated lentivirus, plasmid, and/or shRNA were seeded in 6-well plates and grown to 95%–100% confluence. Next, a scratch was made with a 200 µL pipette tip, and the width of the scratch was measured as the baseline. The width of the scratches was monitored every 12 h using the QCapture Pro software. Finally, the percentage of wound healing was calculated, and the results are shown as the mean ± standard deviation. This assay was repeated three times.

**Transwell assay**

Cell invasion and migration assays were performed using 24-well Transwell plates. The cell invasion assay was performed using 24-well Matrigel invasion chambers with 8-& µ-pore inserts (BD Biosciences) and pre-coated Matrigel (100 μl serum-free medium and 2.6 μl Matrigel, BD Biosciences, San Jose, CA), and the migration assay was performed without pre-coated Matrigel. Next, 5 × 10^5^/ml AGS and SGC-7901 GC cells infected with designated lentivirus, plasmid, and/or shRNA in a 100 μl serum-free medium were seeded into the upper chamber, and a medium containing 20% FBS as a chemotactic agent was added into the lower chamber. After 24 h of incubation, noninvasive cells on the upper membranes were wiped out with a cotton swab. Finally, the cells in the bottom membrane of the upper chamber were fixed in methanol for 30 min and then stained with crystal violet solution for 10 min. This assay was repeated three times.

**Animal experiments**

To explore the effect of BCL6 on GC cell proliferation in vivo, a tumor xenograft model was established in nude mice. Female BALB/c nude mice (4-week-old) were purchased from SPF Biotechnology (Beijing, China). Animal experiments were approved by the Animal Experimentation Ethics Committee of the Tianjin Medical University Cancer Institute. AGS cells [6 × 10^7^/ml, 0.1 ml (50% Matrigel)/mouse, N=8] and SGC-7901 cells (1×10^7^/ml, 0.1 ml/mouse, N=6) stably overexpressing BCL6 and the control lentivirus were injected subcutaneously into the dorsal flanks of 4-week-old female BALB/c nude mice, and tumor growth was monitored regularly. The length (L) and width (W) of the tumor were measured after 22 days, and the tumor volume was calculated as 0.5×L×W^2^. To investigate the effect of BCL6 on the intraperitoneal dissemination of GC cells, SGC-7901 cells overexpressing BCL6 and control lentivirus (1×10^7^/ml, 0.1 ml/mouse, N=12) were injected intraperitoneally into 4-week-old female BALB/c nude mice. Eight weeks after inoculation, the mice were sacrificed and dissected to evaluate peritoneal dissemination, and the weight and number of cancer foci with peritoneal dissemination were determined.

**Immunofluorescence**

Cells were fixed with 4% paraformaldehyde. BCL6 and β-catenin protein were then stained with their antibodies and fluorescent secondary antibodies. Next, the cells were counterstained with DAPI. Finally, the immunofluorescence was visualized and photographed under a fluorescence microscope (Leica).
